# Low-Cost System to Support and Expand Cyanobacterial Harmful Algal Bloom Monitoring with New-Generation Ocean Color Satellites

**DOI:** 10.1021/acsestwater.5c00301

**Published:** 2025-10-24

**Authors:** Chintan B. Maniyar, Keshav Raviprakash, Abhishek Kumar, Mark A. Seferian, Isabella R. Fiorentino, Deepak R. Mishra

**Affiliations:** † Center for Geospatial Research, Department of Geography, 1355 University of Georgia, Athens, Georgia 30602, United States; ‡ College of Engineering, 1355 University of Georgia, Athens, Georgia 30602, United States

**Keywords:** hyperspectral sensor, remote sensing, satellite validation, PACE, Sentinel-3, harmful algal blooms, cyanobacteria, water quality

## Abstract

Cyanobacterial harmful algal blooms (CyanoHABs) pose global risks to public health, ecosystems, and economies. Despite advancements in satellite remote sensing, monitoring gaps persist, particularly in smaller, remote, and resource-limited regions, where CyanoHABs often remain undetected. Satellite-based methods, though effective for large-scale monitoring, suffer from a low spatial resolution, cloud cover, and reliance on in situ validation data. Traditional in situ monitoring equipment, including high-precision spectroradiometers, is costly and logistically challenging, further exacerbating global monitoring inequities. Cyanosense 2.0 (CS2.0) is a low-cost system for real-time in situ CyanoHAB detection and satellite validation designed to address these monitoring voids. CS2.0 integrates two hyperspectral Hamamatsu spectrometers and a microcontroller system, recording Remote Sensing Reflectance (R_rs_) with high agreement to industry-grade instruments (*R* ^2^ = 0.86, Normalized Root Mean Squared Error (NRMSE) = 9.82%), at a fraction of the cost (∼$1300). During field validation in multiple CyanoHAB-prone U.S. lakes, CS2.0 showed a strong performance when tested for widely used satellite-based CyanoHAB models and indices (*R* ^2^ = 0.74–0.83; NRMSE = 13%–18%). The system’s weatherproof design supports long-term autonomous deployments, making it functional in remote environments. As a scalable and accessible solution, CS2.0 holds the potential to democratize CyanoHAB monitoring and improve global water quality assessments, especially in under-represented regions.

## Graphical abstract

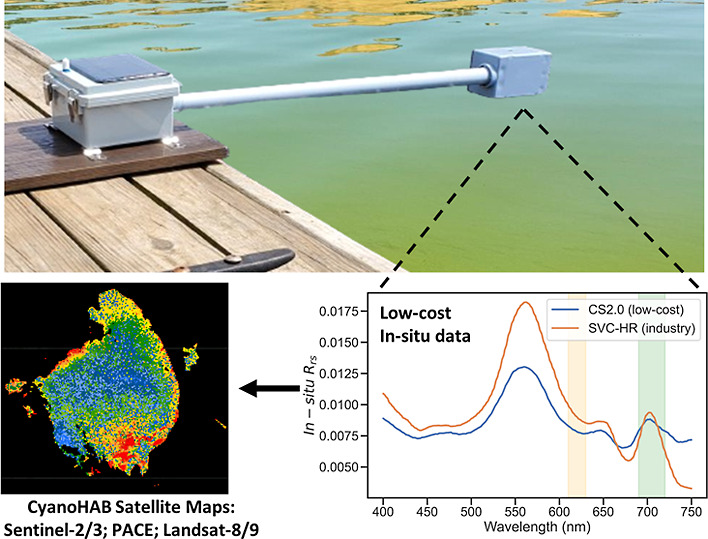

## Introduction

Cyanobacterial harmful algal blooms (CyanoHABs) pose a significant and growing threat to public health, ecosystems, and economies worldwide.^1,2^ These toxic blooms degrade water quality, restrict water use, and cause mass mortality in aquatic life due to hypoxia and toxin exposure.^3,4^ Human exposure to cyanotoxins, such as microcystins and cylindrospermopsins, can lead to severe health problems, including liver damage, neurological disorders, and fatalities, with no known antidotes.^5,6^ Climate change and nutrient pollution have intensified CyanoHAB frequency and severity, particularly in smaller vulnerable water bodies.^7–9^

Despite advancements in solutions for the monitoring and early detection of CyanoHABs, a major challenge remains in the current state of knowledge: the global CyanoHAB monitoring network is heavily biased toward well-funded regions, leaving vast areas under-represented.^10–12^ The limitations of existing monitoring technologies create large data voids, particularly in the Global South, where many water bodies remain unmonitored due to the high cost and logistical challenges of in situ sensors and the coarse spatial resolution of satellites.^13–16^ For example, in 2020, nearly 300 elephants in Botswana died due to suspected cyanotoxin poisoning in isolated watering holes.^17,18^ However, due to the small size and remote location of these water bodies, there was no direct environmental monitoring or conclusive evidence linking the deaths to CyanoHABs.^19,20^ In a more recent instance, a Zimbabwe national park saw an ecological disaster with huge losses in wildlife due to CyanoHAB poisoning in Lake Chivero.^21,22^ Similar unreported ecological and public health crises likely occur worldwide, falling through the cracks of current monitoring systems.^23,24^ Addressing these gaps is critical to understanding the true global extent and impact of the CyanoHABs.

Satellite remote sensing is a powerful tool for large-scale CyanoHAB detection, collaborative risk assessments, and early warning systems.^25–27^ These methods typically leverage spectral signatures of pigments, such as phycocyanin (PC) and chlorophyll-*a* (Chl-*a*).^28–30^ PC, unique to cyanobacteria, exhibits strong absorption at 620 nm and is often used as a proxy for bloom severity and toxicity.^31^ With the advent of new-generation satellite missions, such as Ocean Color Imager (OCI) onboard Phytoplankton, Aerosol, Cloud, ocean Ecosystem (PACE), PRISMA, Surface Biology and Geology (SBG), Landsat Next, etc., newer avenues have opened up for satellite-based water quality monitoring.^32–34^ PACE (OCI) and PRISMA offer the hyperspectral coverage from 340–895 nm at 1.2 km and 30 m spatial resolution, respectively, with high radiometric sensitivity, allowing for accurate pigment retrievals and algorithm development. Several spectral space models, such as the Three-Band PC algorithm (PC_3_)^35^ and the Phycocyanin Index (PCI),^36^ have been developed to estimate PC concentrations. Similarly, Chl-*a*-based indices like the Normalized Difference Chlorophyll Index (NDCI)^37^ and the Cyanobacteria Index (CI)^38^ are widely used for CyanoHAB assessments. These models have been frequently adapted to Sentinel-3′s Ocean and Land Color Imager (OLCI), Sentinel-2’s Multispectral Instrument (MSI), and LandSat series’ Operational Land Imager (OLI) for a continuous large-scale monitoring.^28,39–43^ However, despite their broad coverage, satellite sensors are limited by cloud cover, data gaps, and insufficient spatial resolution. PACE (OCI) and Sentinel-3 (OLCI), for instance, have a 1.2 km and 300 m spatial resolution, respectively, making it inadequate for monitoring small lakes, reservoirs, or localized bloom events.^44–46^ The lack of high-resolution continuous in situ data exacerbates these challenges, limiting the ability to validate satellite-derived bloom estimates and detect blooms in water bodies too small for satellite observation.

In situ monitoring remains crucial for validating satellite models and detecting localized CyanoHABs. High-precision spectrophotometers and spectroradiometers are commonly used for radiometric calibration direct and pigment quantification.^47–49^ The Water Insight Spectrometer (WISP) is another commercial-grade instrument for point-based in situ water quality measurements. However, these instruments are prohibitively expensive and require significant expertise and maintenance, restricting their deployment in remote or resource-limited settings.^50^ Drone-mounted hyperspectral sensors offer a promising alternative for localized mapping but remain costly and logistically challenging.^51–53^ Consequently, CyanoHAB monitoring is disproportionately concentrated in well-funded regions, while many smaller or economically disadvantaged areas lack real-time on-the-ground monitoring.^14,42,45,54^

The emergence of low-cost in situ monitoring technologies offers a potential solution to bridge these data gaps.^12,55–58^ Several budget-friendly systems have been developed, such as portable microbiological toxin detection kits,^59^ optical sensors,^60^ fluorescence-based sensors,^61^ and smartphone-connected spectrophotometers.^62^ Cyanosense, a low-cost spectroradiometer-based system, demonstrated the feasibility of cost-effective in situ monitoring by transmitting radiometric data via a 2G mobile network and relying on solar energy.^54^ However, existing low-cost solutions often face challenges related to data transmission in remote areas, durability under harsh environmental conditions, power limitations in low-light settings, and operational autonomy.^12^

This study introduces Cyanosense 2.0 (CS2.0), an autonomous, low-cost, dual-headed hyperspectral system for real-time and in situ monitoring of CyanoHABs. Assembled using cost-effective components, CS2.0 improves upon its predecessor^54^ by addressing challenges in power efficiency, mechanical durability, and data transmission, making it adaptable to both well-connected and network-void regions. The specific objectives of this study are (a) to design, characterize, and fabricate the CS2.0 system with optimized mechanical durability, power consumption real-time data transmission, and reduced development cost; (b) to validate a CS2.0s radiometric performance against an industry-grade spectroradiometer SVC-HR (SpectraVista Corporation’s HR-1024i) using in situ measurements from freshwater lakes across the United States; and (c) to quantify CS2.0s performance for satellite-based CyanoHAB monitoring by comparing its data with coincident PACE (OCI) and Sentinel-3 (OLCI) data, and using NDCI, PC_3_, CI, and PCI models. CS2.0 is designed to fill monitoring voids by enabling point-to-space CyanoHAB assessments, particularly in under-represented and hard-to-access water bodies. By increasing the accessibility of real-time and localized monitoring technologies, CS2.0 has the potential to democratize CyanoHAB detection and improve global water quality management.

## Materials and Methods

### System Development

The development of CS2.0 aimed to create a low-cost efficient system for monitoring CyanoHABs, building upon its foundational design.^54^ The system integrates electrical, software, and mechanical components to achieve robust field performance while optimizing the power consumption and functionality. Figure 1A shows the overall system architecture for CS2.0. Main components include two Hamamatsu C12880MA spectrometers for spectral intensity measurements, an ESP32 microcontroller for improved processing and storage capabilities, photorelay for signal transmission, and power regulation to the spectrometers. The C12880MA have been used to monitor environment and water quality^55,63^ and were chosen for CS2.0 after evaluations about size, functionality, and cost. The sensors were used with factory-set calibration for spectral response and dark current behavior, with more information about sensor characterization here: https://github.com/cyanotracker/Cyanosense-2.0/blob/main/Documents/Hamamatsu%20Brochure.pdf. Two spectrometers were used for a “dual-sensor” setup, to simultaneously record upwelling and downwelling radiance. These quantities are crucial for remote sensing of CyanoHABs.^64–66^ A Voltaic solar panel and battery pack provided a renewable power source, while data transmission was enabled through a RockBLOCK Iridium 9603 Satellite Modem, which has been previously used for similar applications.^67^ Voltage and current requirements were addressed by using voltage dividers and MOSFETs to adapt the power supply to various system components. An external Real-Time Clock (RTC) module was incorporated to correct timekeeping drifts in the ESP32’s internal RTC, ensuring precise data readings during the autonomous mode.

**Figure 1:**
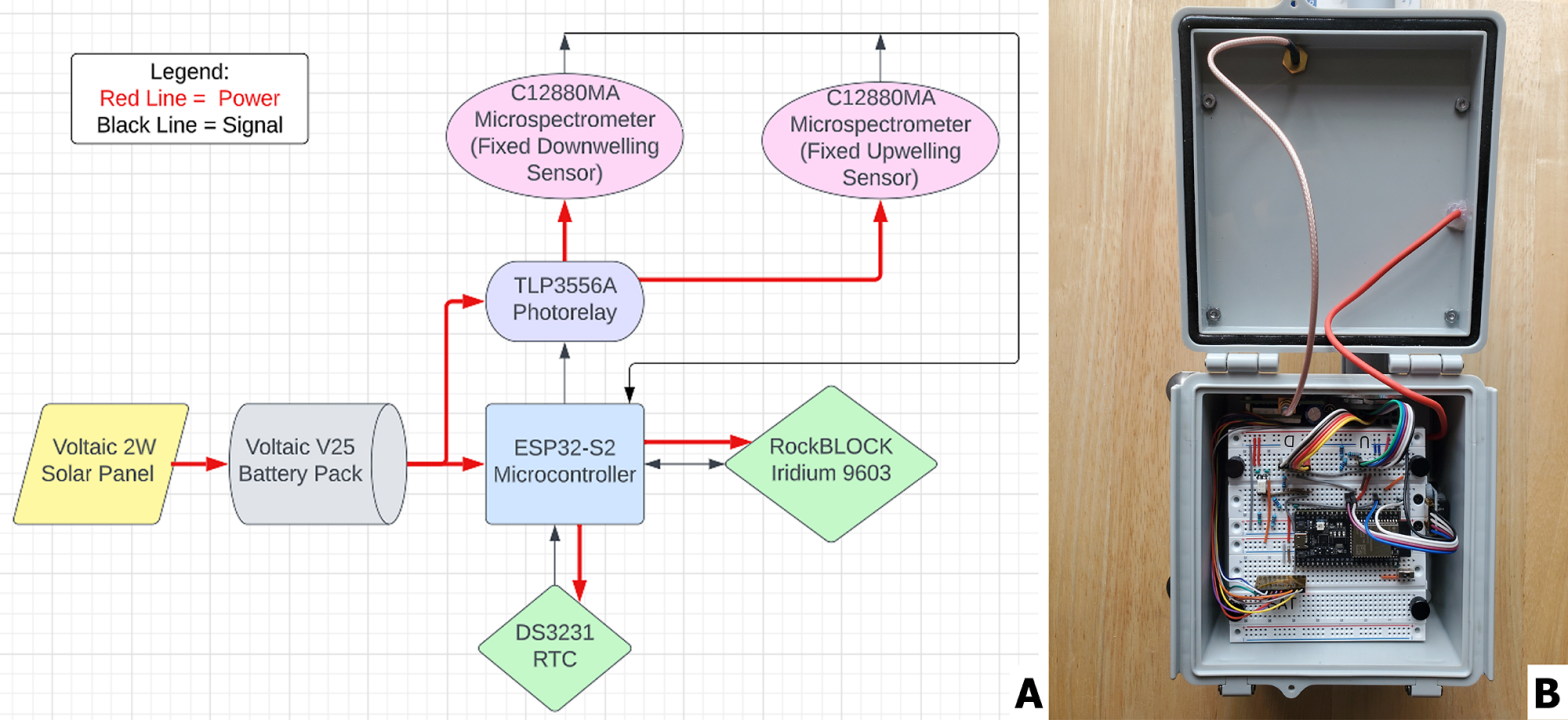
(A) Overall system architecture for CS2.0 encompassing all components used in its development. (B) Picture of electronics housing revealing components connected with color-coded cables, and a waterproof sealing in and around the box lid. Photo by author Mark A. Seferian.

The system housing was adapted from an FSE PVC electric box with waterproof modifications, including silicone-sealed glass lenses and custom 3D-printed mounts for spectrometers. Figure 1B shows the top-view of the electronics housing box, which contains circuitry, satellite modem, and power supply of the system. Waterproofing measures include silicone seals and O-rings for cable entry points. Spectrometers and electronics were connected using a reinforced PVC connector, with additional stability provided by the removable metal feet.

### Study Sites and Data Description

A nationwide field sampling campaign was conducted during the summer (July through October) of 2024 across six lakes in the United States (Figure 2). Sampling locations and dates for each lake were carefully selected using preliminary satellite image analysis, to coincide with ongoing (moderate to severe) CyanoHABs events. Water samples and radiometry data were collected from Green Bay in Wisconsin (July 2024), the Western Basin of Lake Erie in Ohio (Aug 2024), Lake Okeechobee in Florida (Aug 2024), Lake Clear and San Luis Reservoir in California (Sept 2024), and Lake Pontchartrain in Louisiana (Oct 2024). While different in geography, topographic factors, such as depth and size, and trophic status, all six lakes hold a consistent history of severe CyanoHAB events.^1,68–70^

**Figure 2:**
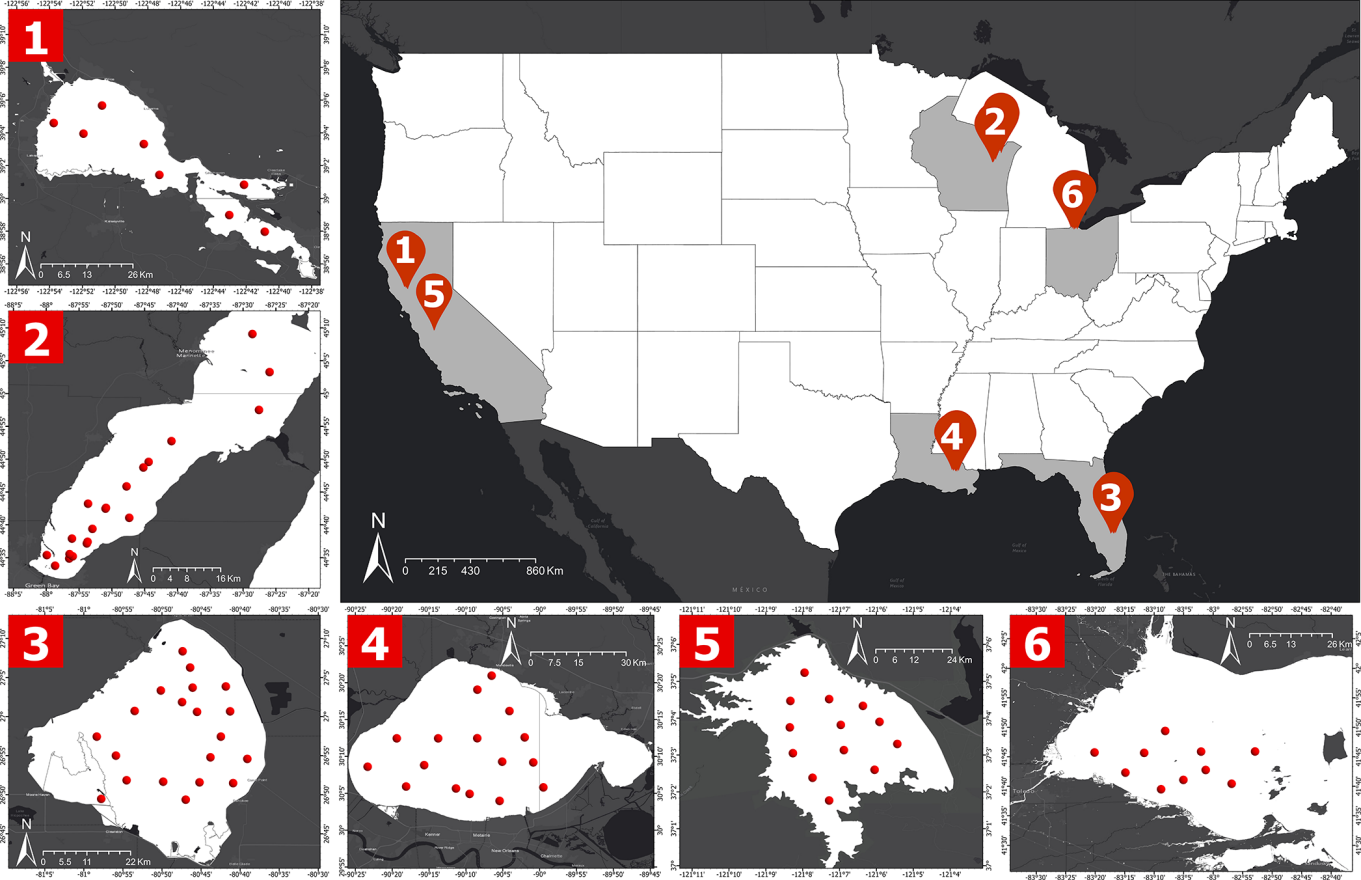
Center: A map showing sampling sites (highlighted in red markers) as six major lakes across the United States, which are also known CyanoHAB hotspots. Inset figures show maps of individual lakes and the red dots show the sampling locations: 1) Clear Lake, California; 2) Green Bay, Wisconsin; 3) Lake Okeechobee, Florida; 4) Lake Pontchartrain, Louisiana; 5) San Luis Reservoir, California; and 6) Lake Erie (Western Basin), Ohio.

Simultaneous radiometric data were collected by using CS2.0 and SVC-HR. SVC-HR is an industry-grade high-precision hand-held hyperspectral spectroradiometer that is widely used in field campaigns for the remote sensing of water, wetlands, and vegetation environments.^71^ Because SVC-HR is also widely used for calibrating airborne and satellite-based spectroradiometers,^72^ it was chosen as an industrial standard to calibrate and validate CS2.0s radiometric performance. For each sampling location, coincident CS2.0 and SVC data was collected. To arrive at remote sensing reflectance (*R*_rs_) using Mobley’s method^66^ (eq 1), three radiometric quantities were recorded: (a) Downwelling or Sky Radiance (*L*_sky_)—amount of light that is incident on the target (water), (b) upwelling or Water Radiance (*L*_w_)—amount of light that is reflected off of the target (water), and (c) diffuse radiance from a Lambertian reflector of known reflectance (*L*_c_) as a reference. A white spectral panel with 99% reflectance was used for this purpose. Data was recorded by aligning CS2.0 and SVC-HR at the same height and while maintaining the same sun-sensor geometry, to ensure ideal conditions for the consequent calibration and validation of R_rs_. Specifically, a sensor zenith angle of 40° off-nadir and a sensor azimuth of 135° (looking back and off the side from sun) to avoid the sun glint was followed, which is consistent with ocean optics best practices to minimize surface reflection and the sun glint effect.^66,73^ CS2.0 has an uplooking and a downlooking spectrometer that record *L*_sky_ and *L*_w_, respectively. *L*_c_ was recorded using a CS2.0's downlooking spectrometer. A cautious protocol was followed to ensure avoidance of any shadows and maintain consistency. External factors, such as sun angle, cloud cover and haze, time of day, were recorded and factored in while processing the data to arrive at an accurate measurement of *R*_rs_.

1 $$R_{rs}=\frac{L_{w}-0.02\times L_{sky}}{\frac{\pi }{R_{ref}}\times 1.01\times L_{c}}$$

where *L*_w_ is the water leaving radiance; *L*_sky_ is the sky radiance and *L*_c_ is the radiance leaving the Spectralon calibration panel (a known reflectance Lambertian surface); and *R*_ref_ is the irradiance reflectance of the reference (0.99, for the 99% white Spectralon panel; ∼0.18 for an 18% gray card).

### Radiometric Data Processing

#### Calibration and Validation of CS2.0 *R*_rs_

After careful QA/QC of all radiometric data, 31 samples across all lakes were determined fit for cross-calibration based on factors, such as cloud cover and solar angles, time difference between CS2.0 and SVC-HR scans, replication consistency, and satellite overpass. CS2.0 records data in 313 nm–889 nm and SVC-HR records data in 331 nm–2500 nm with variable bandwidths. To ensure uniformity, all data were linearly interpolated to 1 nm and CS2.0 and SVC-HR data were aligned to overlap in the wavelength range 390 nm −880 nm, typical for aquatic remote sensing.^74^ CS2.0 records the intensity in Arbitrary Units (AU) across a 6-channel pixel space, which is transformed into the wavelength space using factory-provided calibration coefficients (Section B, Supporting Information). To convert the CS2.0 intensity into physically meaningful radiance units, we performed a wavelength-dependent linear calibration against SVC-HR for *L*_w_, *L*_sky_, and *L*_c_ components. We used a leave-one-location-out validation approach for each lake, where calibration coefficients were derived from five of the six lakes and validated on the sixth lake. The resulting *R*_rs_ was evaluated for magnitude using percentage normalized root mean squared error (%NRMSE), mean absolute percentage error (MAPE), and signed symmetric percentage bias (β). To assess the spectral shape, we used spectral angle mapping (SAM), spectral information divergence (SID), and spectral Euclidean distance (SED).^75,76^ The formulas for these metrics are provided in Section A of the Supporting Information.

#### Upscaling CS2.0 *R*_rs_ for Satellite Validation

To demonstrate CS2.0s capability for point-to-space satellite validation, the hyperspectral OCI sensor onboard NASA’s PACE mission,^77^ and the multispectral OLCI sensor onboard Sentinel-3 are used. A relative spectral response for PACE OCI and Sentinel-3 OLCI (as available at https://oceancolor.gsfc.nasa.gov/resources/docs/rsr_tables/) was used to scale up both CS2.0 and SVC-HR *R*_rs_ to PACE *R*_rs_ and OLCI *R*_rs_, respectively (Figure 3). This resulted in two sets of PACE *R*_rs_—one from CS2.0 (PACE_CS2.0_) and one from SVC-HR (PACE_SVC‑HR_). Similarly, two sets of OLCI *R*_rs_ were obtained—one from CS2.0 (OLCI_CS2.0_) and one from SVC-HR (OLCI_SVC‑HR_). Consequently, four established CyanoHAB spectral indices and models, namely, NDCI,^37^ PC_3_,^35^ CI,^38^ and PCI,^36^ were implemented on the simulated satellite data: both sets of PACE *R*_rs_ and both sets of OLCI *R*_rs_. The performance of satellite *R*_rs_-based CyanoHAB indices and models from PACE_CS2.0_ and OLCI_CS2.0_ was evaluated by considering PACE_SVC‑HR_ and OLCI_SVC‑HR_, respectively, as a reference. Simply put, satellite data simulated from the low-cost system (CS2.0) was validated by considering the satellite data simulated from an industry-grade sensor (SVC-HR) as a reference. Whenever a cloud-free overpass of both PACE and Sentinel-3 was available, spectra from CS2.0, PACE, and Sentinel-3 were compared. Uncertainty in *R*_rs_ and consequent CyanoHAB indices and models was quantified using %NRMSE, MAPE, and β, metrics commonly used in spectral evaluations.^1,2,37,74,78^ The equations and formulas for CyanoHAB indices/models and error metrics are described in Section A of Supporting Information.

**Figure 3:**
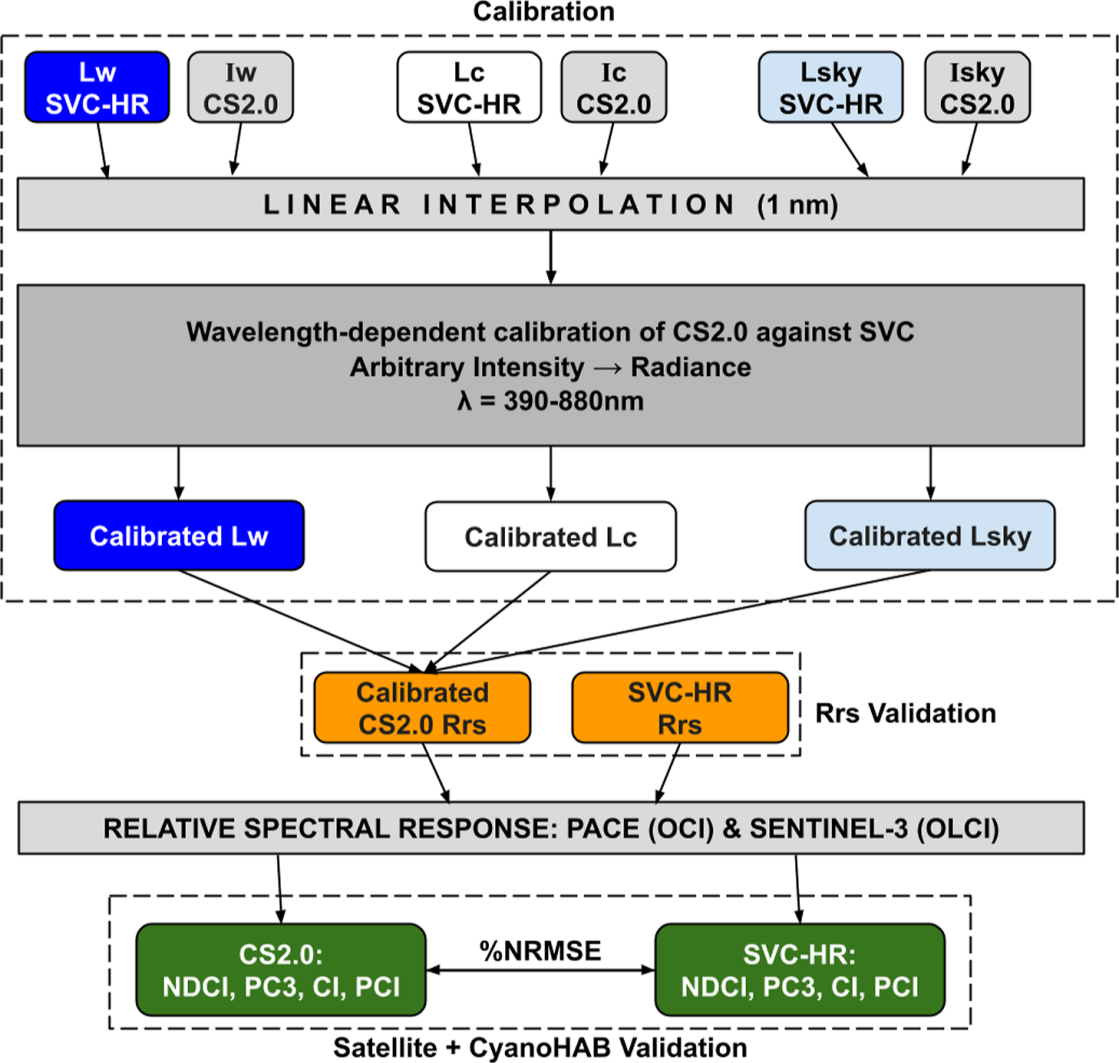
Overall methodology showing a stepwise process of calibrating and validating *R*_rs_ from CS2.0 against SVC-HR, and consequent satellite scaling to PACE (OCI) and Sentinel-3 (OLCI), followed by implementation of CyanoHAB specific indices on simulated satellite *R*_rs_.

## Results and Discussion

### System Development and Field Deployment

CS2.0 builds on the foundational design of its predecessor, Cyanosense,^54^ incorporating advancements that improve durability, functionality, and operational efficiency. The system has a robust mechanical structure suitable for field conditions, addressing common challenges in low-cost in situ water quality monitoring.^12^ Figure 4A shows the completed CS2.0 design. The advancements and upgrades were achieved without compromising overall cost-efficiency–CS2.0 was developed at a low cost of ∼$1,300, which is cheaper than its parent system,^54^ and just a fraction of the expense associated with industry-grade spectroradiometers.^50^ Section D, Table 2 of the Supporting Information lists the components and the development budget of CS2.0 in greater detail. All necessary data and documentation to rebuild/replicate CS2.0 can be found at https://github.com/cyanotracker/Cyanosense-2.0/tree/main.

**Figure 4:**
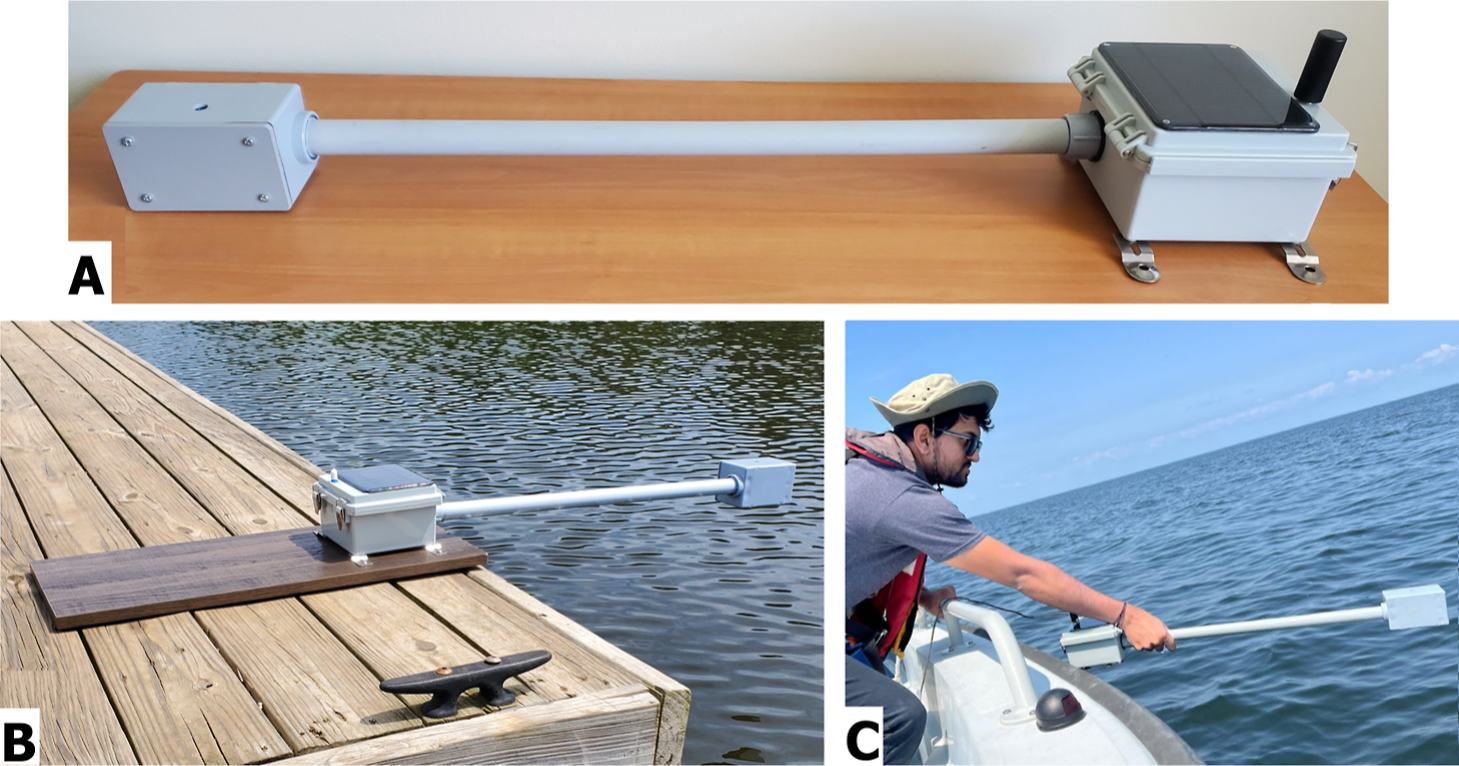
(A) Complete CS2.0 design post final assembly. The two spectrometers are in the system casing on the left end, one looking up and the other looking down. The right end of the system is the electronics housing, with the solar panel and antenna for satellite data transmission. Photo by author Mark A. Seferian. (B) Autonomous operation: CS2.0 deployed at Lake Herrick in Georgia, operating and transmitting data autonomously. Photo by author Mark A. Seferian. (C) Manual operation: Author Chintan B. Maniyar using CS2.0 as a hand-held system to record radiometric data at the Green Bay of the Lake Michigan, from onboard a research vessel. Photo by author Abhishek Kumar.

The system’s overall structure did not comprise any moving parts or loose components, thereby improving mechanical longevity compared to similar low-cost systems.^56,79^ CS2.0 uses the Iridium satellite network for data transmission, mitigating a common issue in low-cost remote sensing systems that often require frequent on-site visits for troubleshooting and manual data retrieval.^12,80^ Iridium is known to offer reliable operation in remote network-limited regions,^81^ where CyanoHABs often may go undetected. Additionally, the power efficiency is a major limitation in existing systems, often restricting long-term deployments to only a few days without solar charging.^54^ CS2.0 is powered via a system battery as well as a solar panel. Power management is optimized through timed energy harvesting, allowing for sustained operation for up to 25 days in the absence of solar charging. Field tests indicate a net battery gain of 763.04 mAh per day, with a total power draw of 256.96 mAh and a power return of 1020 mAh under standard solar charging conditions (Section F, Figure 2, Supporting Information). To our knowledge, this makes CS2.0 one of the most power-efficient water quality monitoring systems yet.^12,14,80^ CS2.0's weatherproof and temperature-proof designs maintained the electronics housing temperature within an optimal range for functionality,^82^ with spectrometer temperatures reaching 32.6 °C at an ambient temperature of 23.7 °C (Section E, Tables 3 and 4, Supporting Information). Higher temperatures were occasionally noted to increase the dark current magnitude, which was reflected in sensor calibration biases. Figure 4B shows the deployment mode of CS2.0 at Lake Herrick, Georgia, wherein it autonomously transmits radiometric data.

CS2.0 is also designed for manual operation as a hand-held system suitable for field visits to water sites. Spectrometers mounted on an extended PVC pipe minimize the shadow interference and help maintain optimal azimuth and zenith angles. CS2.0 was operated as a hand-held system at all six of our study sites during field sampling. Figure 4C demonstrates its use in the hand-held mode from a research vessel at Green Bay, Lake Michigan.

### Radiometric Calibration and Validation of CS2.0 *R*_rs_

Leave-one-location-out analysis showed strong generalization and transferability for all radiance components: *L*_w_—water with *R* ^2^ = 0.91, (*p* < 0.001); *L*_c_—spectralon calibration panel with *R* ^2^ = 0.86, (*p* < 0.001); *L*_sky_—sky with *R* ^2^ = 0.92, (*p* < 0.001) (Figure 5A–C); as well as *R*_rs_: *R* ^2^ = 0.86, *p* < 0.001 (Figure 5D). Figure 1, Section B of the Supporting Information for a site-by-site CS2.0 and SVC-HR *R*_rs_ spectra overlay and comparison. This resulted in wavelength-specific coefficients and biases for each component. We have described these coefficients and biases in greater detail in Table A of Supporting Information, which can be used to calibrate CS2.0. These calibration coefficients are appropriate for newly built CS2.0 systems, however, to assess the spectrometer drift over time, the users should perform fresh calibration. In an attempt to not let this limit usability, free of charge recalibration services would be offered upon contact from the users.Supporting Information

**Figure 5:**
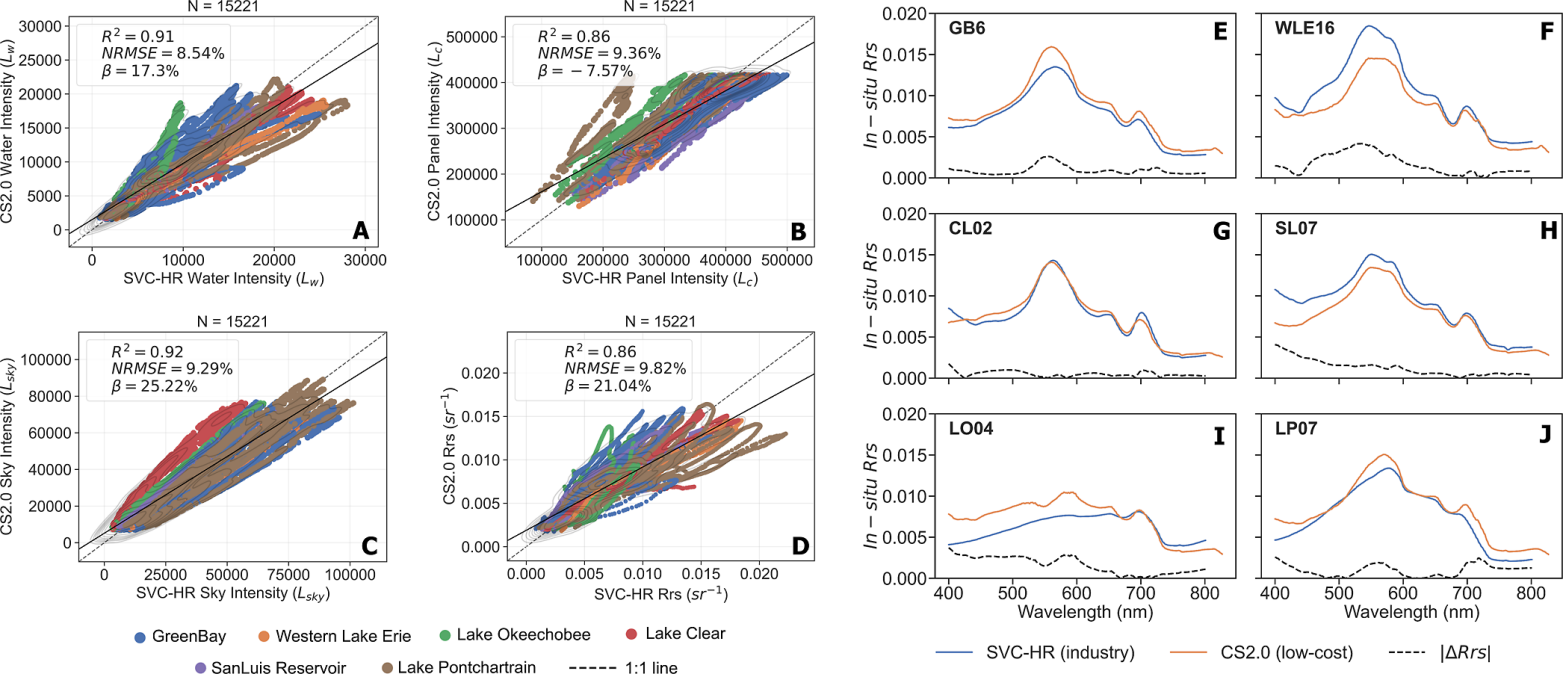
Validation of CS2.0 radiance against SVC-HR radiance for three components used in *R*_rs_ computation across the 390–880 nm wavelength range: *L*_c_—calibration panel (A), *L*_sky_—sky (B), and *L*_w_—water (C). (D) Validation fit of calibrated *R*_rs_. Each plot includes *R* ^2^, %NRMSE, and Bias (β) metrics. Saturation in *L*_c_ (A) is confined to wavelengths in the range 460 nm–545 nm and does not affect *R*_rs_ of CyanoHAB specific bands used in this study. Data shown are from six study sites: Green Bay (WI), Western Lake Erie (OH), Lake Okeechobee (FL), Clear Lake (CA), San Luis Reservoir (CA), and Lake Pontchartrain (LA). Radiometric data were linearly interpolated to 1 nm resolution. The solid black line represents the validation fit, and the dotted gray line represents the 1:1 reference line. Right panel (E–J): Comparison of calibrated in situ *R*_rs_ from CS2.0 (blue) with in situ *R*_rs_ from SVC-HR (orange). The dotted black line indicates the absolute error. One representative set of spectra is shown for each site: (E) Green Bay, WI; (F) Western Lake Erie, OH; (G) Clear Lake, CA; (H) San Luis Reservoir, CA; (I) Lake Okeechobee, FL; and (J) Lake Pontchartrain, LA.

The Hamamatsu spectrometers exhibited saturation effects under high-intensity conditions, resulting in a symmetric positive bias in *L*_sky_ and *L*_w_, and a negative bias in *L*_c_. Consequently, the CS2.0-derived *R*_rs_ showed approximately 21% bias, relative to SVC-HR measurements. Figure 5E–J shows in situ *R*_rs_ spectra from CS2.0 (in blue) and SVC-HR (in orange) overlaid onto each other and absolute error (in dashed black); one from each of the six sampling lakes. The bias in CS2.0-derived *R*_rs_ often manifested as slightly depressed reflectance peaks in the spectra, particularly in the 710 nm region. This is illustrated in Section B, Figure 1 of the Supporting Information), which provides a set of both CS2.0 and SVC-HR *R*_rs_ spectra from all sampling sites.

The evaluation of *R*_rs_ showed a generally high agreement in terms of the spectral magnitude and shape with average %NRMSE and MAPE ∼20%, and an absolute bias less than 5%, for all wavelengths. To evaluate the spectral magnitude of CS2.0 *R*_rs_, %NRMSE and MAPE were calculated for each wavelength across all 31 samples, using SVC *R*_rs_ as the reference. Figure 6A shows how the error varies across the 390 nm–880 nm wavelength range, with the CyanoHAB active region^30^ highlighted in green. Shorter and longer wavelengths showed higher errors due to the instrument edge effect—a known issue in spectro-radiometric instruments.^83,84^ The edge effects usually manifest as spikey or low SNR signals, typically due to the reduced efficiency and sensitivity of spectrometer components in the extremes of its operational range. The region around 710 nm showed a high error, indicating a subdue effect or decreased sensitivity by the Hamamatsu spectrometers for that region. Similar instrument artifacts have been noted before, when comparing low-cost systems with high-precision systems.^85^ To evaluate the spectral shape—an important factor, as an accurate shape can still enable the use of ratio-based and spectral shape-based remote-sensing algorithms even when *R*_rs_ magnitude is suboptimal—we used SAM, SID, and SED metrics^49,75^ (see the Supporting Information, Section A). All 31 samples showed low values for these metrics, indicating a generally high agreement in the spectral shape (Figure 6B). The current performance makes CS2.0 suitable for CyanoHAB monitoring as most widely used remote-sensing CyanoHAB models are based on *R*_rs_.^2,28,30,86^

**Figure 6:**
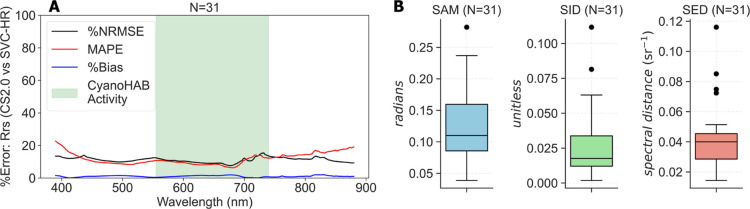
Evaluation of CS2.0 *R*_rs_ with SVC-HR *R*_rs_ as a reference for: (A) magnitude using %NRMSE, MAPE, and absolute bias metrics, with the shaded green region showing a CyanoHAB active wavelength region and (B) spectral shape using SAM, SID, and SED metrics. Metrics shown are for all 31 samples and span the wavelength range of 390 nm–880 nm.

### Upscaling CS2.0 *R*_rs_ for Satellite Validation

CS2.0 showed a strong correlation with SVC-HR when used for the simulated satellite-scale validation of CyanoHAB monitoring. *R*_rs_ data from CS2.0 were spectrally upscaled to match the configurations of the PACE (OCI) and Sentinel-3 (OLCI) satellite sensors (denoted PACE_CS2.0_ and OLCI_CS2.0_, respectively) and validated against concurrent SVC-HR measurements, similarly upscaled (denoted PACE_SVC‑HR_ and OLCI_SVC‑HR_). To evaluate CS2.0s potential for operational satellite-based CyanoHAB monitoring, four established indices, NDCI, PC_3_, CI, and PCI, were computed using the simulated satellite data. CS2.0-derived satellite *R*_rs_ data at the spectral bands relevant to these indices (560, 620, 665, 681, and 708 nm for PACE; Bands 6, 7, 8, 10, and 11 for OLCI) yielded an average %NRMSE of approximately ∼22% for both PACE and OLCI (Figure S2, Section C of the Supporting Information). Figures S3 and S4 of Supporting Information further illustrate satellite-scaled spectra from CS2.0 overlaid with SVC-HR spectra along with corresponding relative error plots for all sampling stations.

NDCI, PC_3_, CI, and PCI indices from PACE_CS2.0_ and OLCI_CS2.0_ showed a high correlation with their SVC-HR-based counterparts (*R* ^2^ = 0.74–0.83; *p*< 0.001) and relatively low %NRMSE (14–18%), with a negligible bias (Figure 7). Section C, Table 1 of the Supporting Information shows a summary of evaluation metrics for CyanoHAB indices and models as well as the corresponding bands used for each index/model. The average %NRMSE for all indices and models was comparable between sensors (PACE OCI: ∼15.47%, Sentinel-3 OLCI: ∼16.51%).

**Figure 7:**
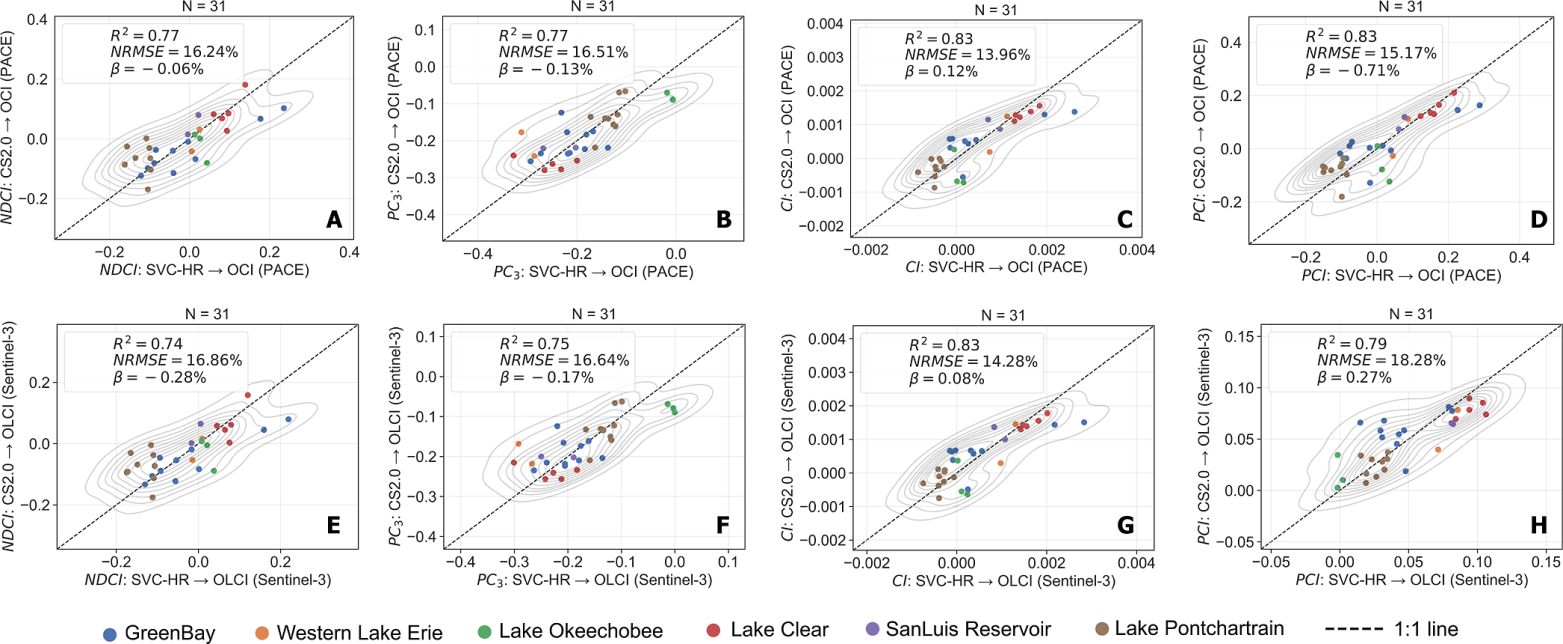
Top row (A–D): Validation of PACE_CS2.0_-derived CyanoHAB indices/models with PACE_SVC‑HR_-derived CyanoHAB indices/models as a reference; with (A) as NDCI, (B) as PC_3_, (C) as CI, and (D) as PCI. Bottom row (E–H): Validation of OLCI_CS2.0_-derived CyanoHAB indices/models with OLCI_SVC‑HR_-derived CyanoHAB indices/models as a reference; with (E) as NDCI, (F) as PC_3_, (G) as CI, and (H) as PCI. A vicarious calibration was performed to overcome over/underestimation of all indices derived from PACE_CS2.0_ and OLCI_CS2.0_. Dotted black line is the reference (1:1) line. Samples from different lakes are shown in different colors.

Joint CyanoHAB monitoring with CS2.0 and satellite data from PACE (OCI) and Sentinel-3 (OLCI) showed consistency between CS2.0 in situ and satellite spectra. Cloud-free overpasses of both satellite sensors, coincident with CS2.0 data collection, were available at eight sampling locations across Green Bay, Lake Erie, Clear Lake, and Lake Pontchartrain. Satellite data were atmospherically corrected using the ACOLITE tool,^88,89^ as available at https://github.com/acolite/acolite. Figure 8 shows coincident in situ (CS2.0) and satellite (PACE and Sentinel-3) spectra overlaid together. Differences in the spectral magnitude, particularly between the satellite and in situ measurements, are attributed to atmospheric effects present in satellite observations but absent in *in situ* CS2.0 data.

**Figure 8:**
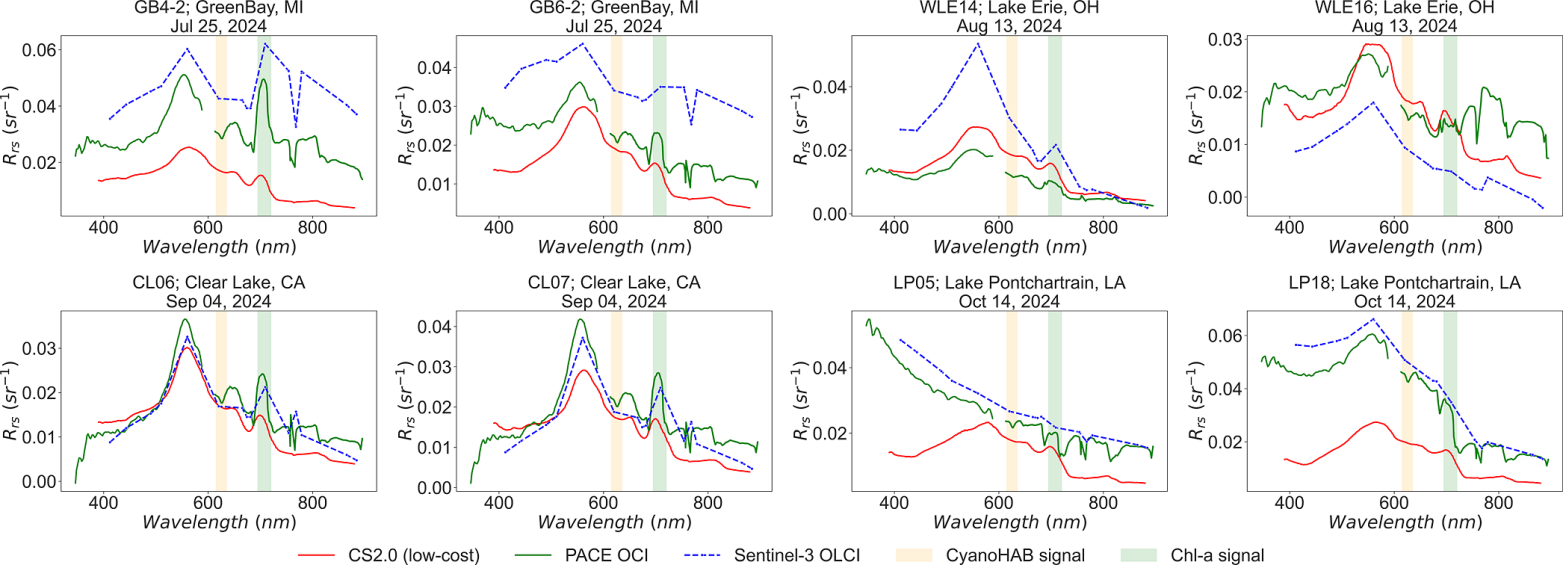
Site-by-site comparison of in situ CS2.0 *R*_rs_ (red) with coincident satellite-derived *R*_rs_ from PACE (green) and Sentinel-3 (blue). For PACE OCI, the 590–610 nm region is excluded in accordance with NASA Ocean Color OB.DAAC guidelines.^77,87^ CyanoHAB-sensitive wavelength regions are highlighted in yellow (620 nm dip) and green (710 nm peak).

These eight representative examples align with the patterns observed in the simulated satellite data (Figure 7). Across the study sites, Green Bay and Clear Lake exhibited the highest PC and Chl-*a* concentrations (higher NDCI, CI and PCI values; lower PC_3_ values), while Lake Pontchartrain and Lake Okeechobee showed the lowest concentrations (Figure 7). The same is also evident in the actual satellite spectra, as seen in the absorption dip around 620 nm and reflectance peak around 710 nm in the satellite-*in situ* overlay plots (Figure 8A,B for Green Bay; (E,F) for Clear Lake, and (G,H) for Lake Pontchartrain). Collectively, these results demonstrate that CS2.0 can effectively capture cyano-sensitive spectral features, making it suitable for acquiring ground truth or match-up data to support ocean color satellite missions as well as for developing or fine-tuning satellite-based models.

### Implications for CyanoHAB Monitoring Programs

CS2.0 can support existing CyanoHAB monitoring frameworks by accompanying real-time or long-term autonomous deployment activities, increasing the temporal resolution of field measurements and satellite match-up capacity. It can also engage the community participation for local-scale monitoring,^1^ helping to close persistent spatial and temporal gaps in the global CyanoHAB assessment.^13,14,33^ While CS2.0 is not yet designed to meet regulatory-grade specifications for accuracy and precision at this stage, it can complement a range of efforts, from federal initiatives like NOAA’s CyAN,^90^ the EPA’s National Lakes Assessment,^91,92^ and USGS sensor networks^93^ to citizen science and low-cost DIY sensor deployments.^54^ Overall, CS2.0 can serve as an accessible and scalable tool for enhancing CyanoHAB situational awareness and prioritizing locations for a more detailed regulatory follow-up.

## Conclusions

This study presents the development and validation of CS2.0, a low-cost autonomous hyperspectral system designed to support CyanoHAB monitoring in developing and under-represented regions. Its solar-powered operation and satellite-based data transmission make it viable for long-term deployment in remote or no-network regions. Field evaluations across diverse water bodies in the U.S. showed a high-measurement accuracy under varying weather conditions and geographies, with a strong agreement with an industry-grade SVC-HR spectroradiometer (*R*_2_ = 0.86 for *R*_rs_). Beyond the in situ use, CS2.0 was tested for joint monitoring with ocean color satellites. CS2.0-derived CyanoHAB indices (NDCI, PC_3_, PCI, and CI) showed a favorable performance (% NRMSE = 14–18%, *R* ^2^ = 0.74–0.83) on simulated PACE and Sentinel-3 satellite data. Comparisons with coincident real PACE and Sentinel-3 overpasses showed a consistent spectral agreement. This compatibility positions CS2.0 as a flexible platform for current and upcoming missions, such as Landsat 8/9/10,^34^ GLIMR, and SBG.^32^

Looking ahead, further improvements could focus on developing robust self-calibration protocols, improving sensitivity in the far-red and NIR region (∼710 nm) to better capture extreme bloom events; and on integrating additional environmental sensors (temperature, pH, and dissolved oxygen) for a more holistic water quality monitoring. Operating over 390–880 nm, CS2.0 is also suitable for broader aquatic applications beyond CyanoHABs. Overall, this work highlights the feasibility and utility of low-cost autonomous hyperspectral systems for scalable, accessible, and equitable environmental monitoring.

## Supplementary Material



## Data Availability

All schematics, design files, and code needed to replicate and reproduce the system are available on the following GitHub repository: https://github.com/cyanotracker/Cyanosense-2.0. In situ data (radiometry, pigment concentrations and coordinates) are available from the first author (CBM) upon request. The code related to data analysis presented in this study will be released in the same GitHub repository.

## Notes

Contents: A) CyanoHAB models and indices; and uncertainty metrics, B) Calibration coefficients for *L*_w_, *L*_c_, and *L*_sky_, C) Upscaling CS2.0 to satellite spectral configuration: radiometric evaluation, D) List and price of components, E) Heatproof testing on field, F) Power budget from field operation (PDF)

## §

AI Institute for Advances in Optimization, Georgia Institute of Technology, Atlanta, GA, 30332, USA
