# Supplementary material for: Low-Cost System to Support and Expand Cyanobacterial Harmful Algal Bloom Monitoring with New-Generation Ocean Color Satellites
Source: ACS ES T Water. 2025 Oct 24;5(11):6246–57. doi: 10.1021/acsestwater.5c00301 (PMC12624730; doi:10.1021/acsestwater.5c00301)
Supplement: Supplementary file 1 [file ew5c00301_si_001.pdf]

Supporting Information for:

Low-cost System to Support and Expand CyanoHAB Monitoring with New Generation Ocean Color Satellites

Chintan B. Maniyar[1*], Keshav Raviprakash[1,2], Abhishek Kumar[1], Mark A. Seferian[2,3], Isabella R. Fiorentino[1] and Deepak R. Mishra[1]

[1]Center for Geospatial Research, Department of Geography, University of Georgia, Athens, GA, 30602, USA
[2]College of Engineering, University of Georgia, Athens, GA, 30602, USA
[3]AI Institute for Advances in Optimization, Georgia Institute of Technology, Atlanta, GA, 30332, USA

**corresponding author email: chintanmaniyar@uga.edu*

This document provides additional equations, figures and tables as supporting information for the article mentioned above, with the following sections:

A. CyanoHAB Models and Indices; Uncertainty Metrics
B. Calibration Coefficients for L_w, L_c and L_{sky}
C. Upscaling CS2.0 to Satellite Spectral Configuration: Radiometric Evaluation
D. List of Price and Components
E. Heatproof Testing on Field
F. Power Budget from Field Operation

Low-cost System to Support CyanoHAB Monitoring with New Generation Ocean Color Satellites: Supporting Information

A GitHub repository containing all designs, schematics, bill of materials and documentation necessary to reproduce and replicate Cyanosense2.0 sensor can be found here: *https://github.com/cyanotracker/Cyanosense-2.0*

A video demonstrating operation of Cyanosense2.0 can be found here: *https://youtu.be/9sZjeJtRidM*

A. CyanoHAB Models and Indices; and Uncertainty Metrics

$$NDCI = \frac{R_{rs}(708) - R_{rs}(665)}{R_{rs}(708) + R_{rs}(665)} \quad (1)$$

$$PC_3 = (R_{rs}^{-1}(620) - R_{rs}^{-1}(665)) \times R_{rs}(708) \quad (2)$$

$$CI = -1 \times \{ R_{rs}(681) - R_{rs}(665) - (R_{rs}(708) - R_{rs}(665)) \times \frac{681-665}{708-665} \} \quad (3)$$

$$PCI = R_{rs}(560) - R_{rs}(620) + (R_{rs}(665) - R_{rs}(560)) \times \frac{620-560}{665-560} \quad (4)$$

where $R_{rs}(560)$ and corresponds to OLCI's band-6; $R_{rs}(620)$ corresponds to OLCI's band-7; $R_{rs}(665)$ corresponds to OLCI's band-8; $R_{rs}(681)$ corresponds to OLCI's band-10; $R_{rs}(708)$ corresponds to OLCI's band-11. These bands are available on PACE OCI verbatim.

A.1 Uncertainty Metrics for Model Validation and Spectral Magnitude Evaluation

$$\%NRMSE = \sqrt{\sum_{i=1}^{n} \frac{(p_i - p_i')^2}{n}} \times \frac{1}{p_{max} - p_{min}} \times 100\% \quad (5)$$

$$MAPE = \frac{1}{n} \sum_{i=1}^{n} \frac{|p_i - p_i'|}{p_i} \times 100\% \quad (6)$$

$$\beta = 100 \times sgn\left(median\left(log_e\left(\frac{p_i'}{p_i}\right)\right)\right) \times \left(e^{\left|median\left(log_e\left(\frac{p_i'}{p_i}\right)\right)\right|} - 1\right) \quad (7)$$

where p is the reference value (SVC-HR); p' is the measured value (CS2.0); n is the total number of samples.

A.2 Uncertainty Metrics for Spectral Shape Evaluation

$$SAM = cos^{-1}\left(\frac{Rrs_{CS2.0} \cdot Rrs_{SVC}}{||Rrs_{CS2.0}|| \; ||Rrs_{SVC}||}\right) \quad (8)$$

$$SID(p, q) = \Sigma_i \; p_i' \, log(\frac{p_i'}{p_i}) + p_i \, log(\frac{p_i'}{p_i}) \quad (9)$$

$$SED = \sqrt{\sum_{i=1}^{N}(Rrs_{CS2.0} - Rrs_{SVC})^2} \qquad (10)$$

where p is the reference value (SVC-HR); p' is the measured value (CS2.0); N is the total number of wavenumbers (wavelength).

B. Calibration Coefficients for L_w, L_c *and* L_{sky}

The Hamamatsu C12880MA spectrometer records data in a 6-channel pixel space, which is converted to wavelength space using coefficients provided by the manufacturer as found in this document. Once in wavelength space, the data is still in Arbitrary Units (AU), representing Intensity. To convert this intensity into various radiances (L_w, L_c and L_{sky}); we provide a wavelength-dependent set of coefficients and biases below for each component for the wavelength range 390nm – 880nm. This table can be found at the end of this document due to its length. Figure 1 shows the Rrs spectra from CS2.0 overlaid with coincident Rrs spectra from SVC-HR, following leave-one location out cross-validation, for all stations across the 6 study sites (lakes).

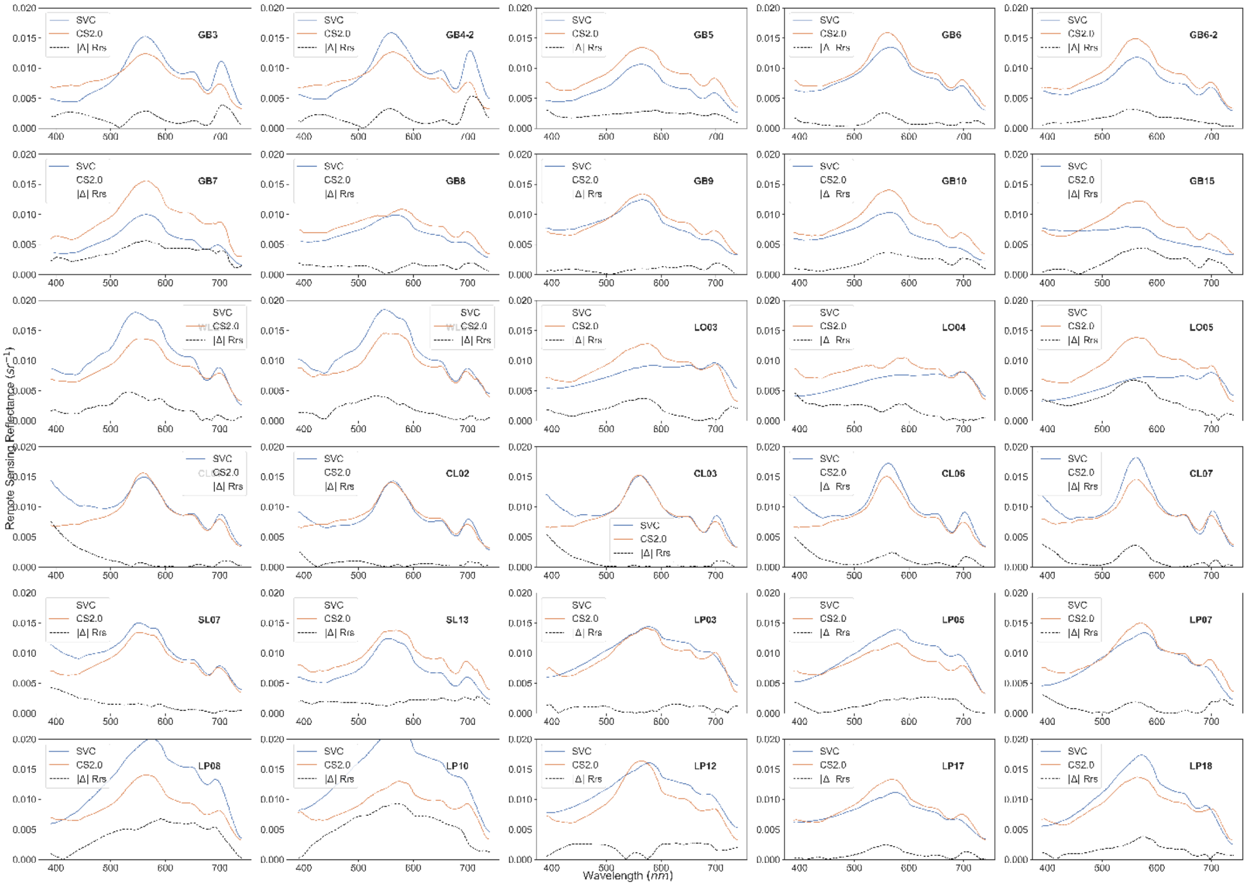

Figure 1: In-situ Rrs spectra of CS2.0 and SVC overlayed onto each other for all sampling stations. Dotted line represents absolute error in Rrs for CS2.0 spectra with SVC spectra as reference.

C. Upscaling CS2.0 to Satellite Spectral Configuration: Radiometric Evaluation

Table 1: A summary of evaluation metrics for - (Top half): CyanoHAB specific bands in PACE (OCI) as well as Sentinel-3 (OLCI). Satellite R_{rs} simulated from CS2.0 is evaluated against satellite R_{rs} simulated from SVC-HR; and (Bottom half): CyanoHAB indices/models applied on PACE (OCI) as well as Sentinel-3 (OLCI). Satellite indices/models simulated from CS2.0 are evaluated against the corresponding ones simulated from SVC-HR.

	%NRMSE and MAPE		R^2		Band Usage
	PACE (OCI)	Sentinel-3 (OLCI)	PACE (OCI)	Sentinel-3 (OLCI)	
		CyanoHAB Specific Bands			
560 nm	23.98% & 22.97%	24.00% & 22.80%	0.45	0.34	PCI
620 nm	21.62% & 20.33%	21.63% & 20.33%	0.41	0.40	PC_3, PCI
665 nm	20.73% & 19.39%	20.88% & 19.51%	0.55	0.51	NDCI, PC_3, CI, PCI
681 nm	19.92% & 18.34%	19.83% & 18.34%	0.58	0.61	CI
708 nm	22.6% & 24.19%	22.66% & 24.19%	0.32	0.38	NDCI, PC_3, PCI
		CyanoHAB Specific Spectral Indices/Models			
NDCI	16.24%	16.86%	0.77	0.74	-
PC_3	16.51%	16.64%	0.77	0.75	-
CI	13.96%	14.28%	0.83	0.83	-
PCI	15.17%	18.28%	0.83	0.79s	-

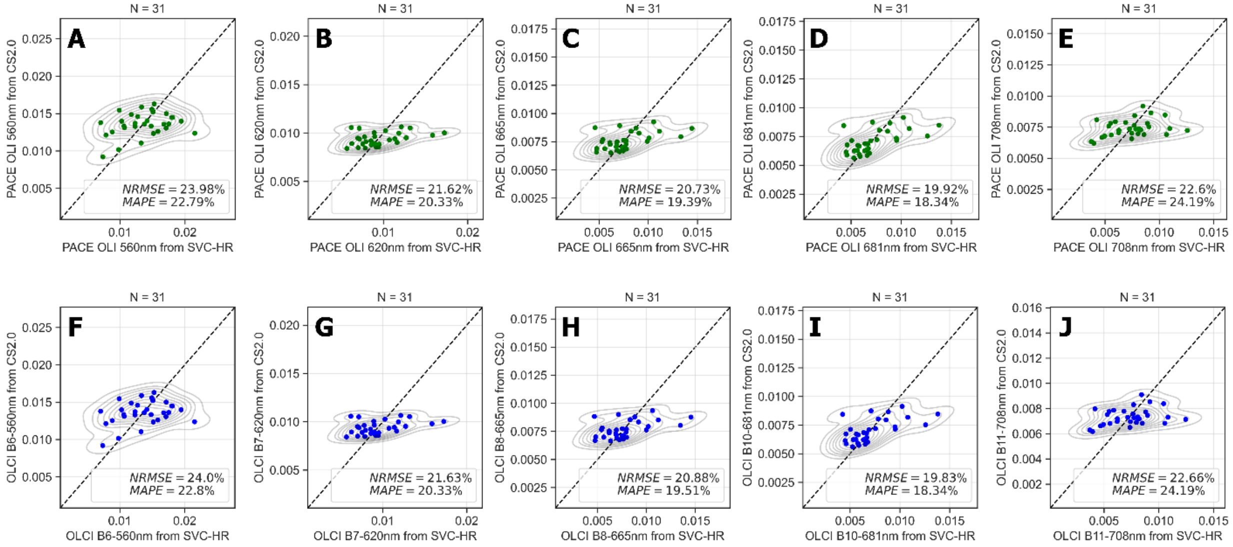

Figure 2: Top row (A-E): Validation of PACE {CS2.0} simulated Rrs with PACE {SVC-HR} simulated Rrs as reference (shown in green). Bottom row (F-J): Validation of OLCI {CS2.0} simulated Rrs with OLCI {SVC-HR} simulated Rrs as reference (shown in blue). Dotted line is the reference (1:1) line.

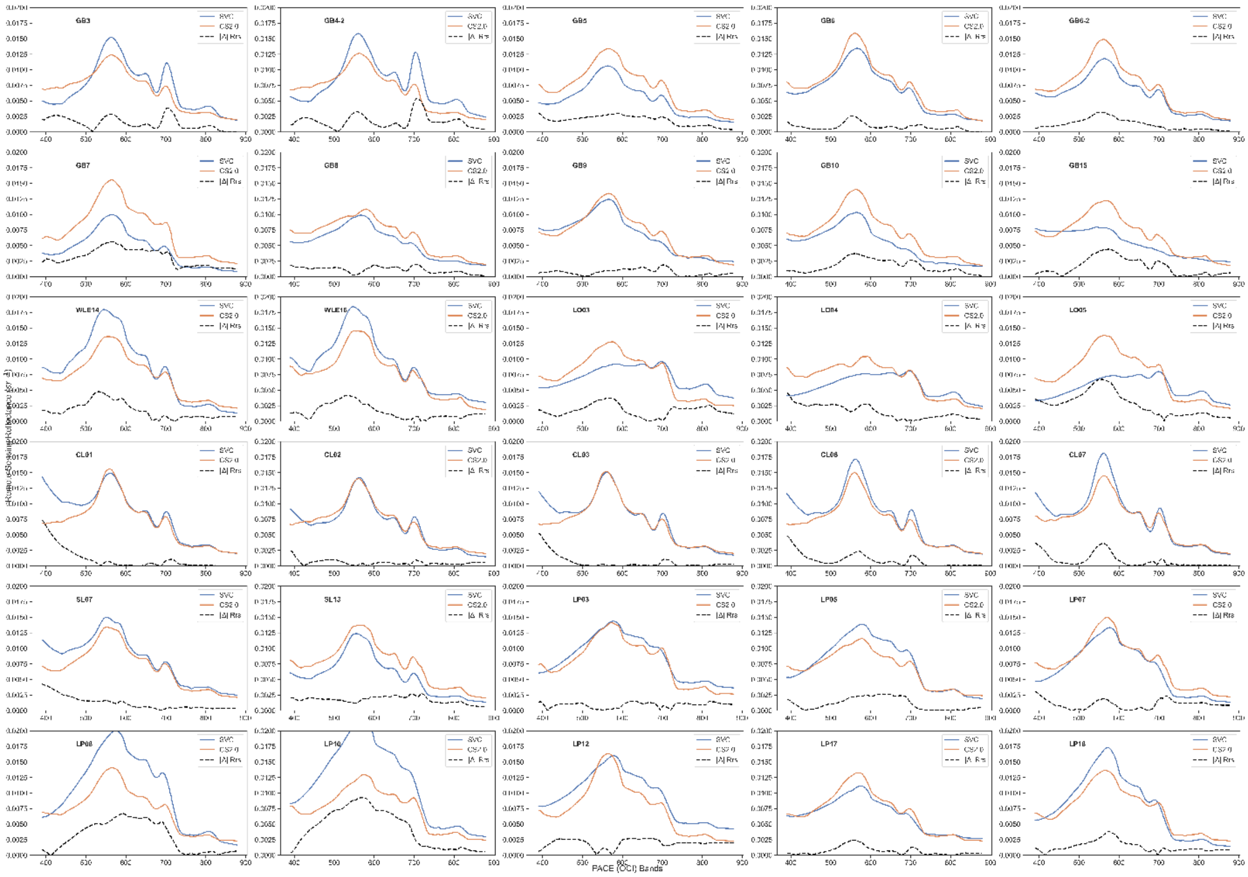

Figure 3: PACsE (OCI) spectral shape derived from PACE$_{CS2.0}$ Rrs (in orange) overlayed onto the corresponding spectral shape derived from PACE$_{SVC\text{-}HR}$ Rrs (in blue) for all sampling stations. Dotted line is the relative error.

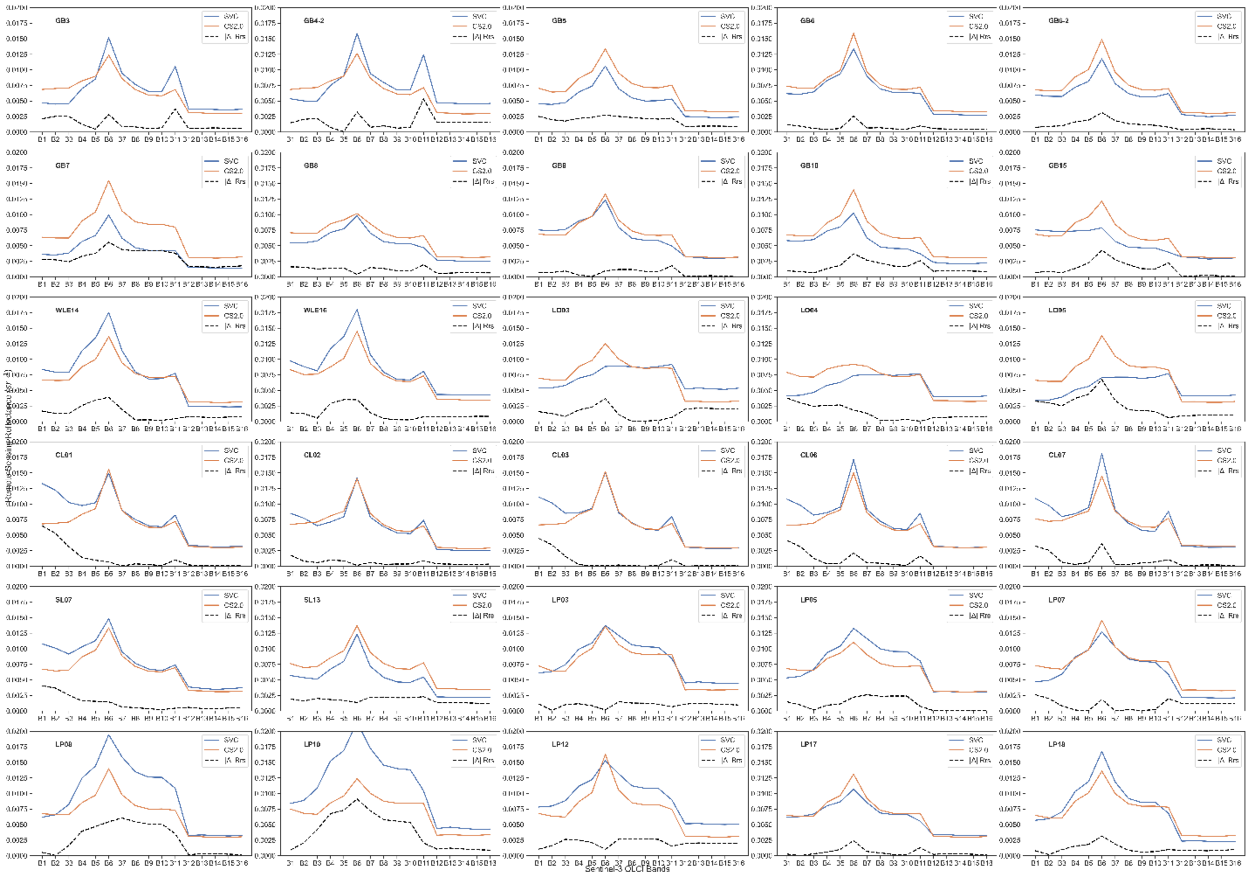

Figure 4: Sentinel-3 (OLCI) spectral shape derived from Sentinel-3$_{CS2.0}$ Rrs (in orange) overlayed onto the corresponding spectral shape derived from Sentinel-3$_{SVC-HR}$ Rrs (in blue) for all sampling stations. Dotted line is the relative error.

D. List and Price of Components

Table 2 includes a comprehensive list of all the components used in the development of CS2.0, along with their price listings. This table also serves as a budget table for CS2.0. Each component name carries the hyperlink to its online listing that was used to make the purchase at the time of sensor development. Note that the prices are based on the EXACT number to build the prototype, unless noted otherwise. Tax and any shipping costs are NOT included. The total cost to build CS2.0 was $1,300.33.

Table 2: A comprehensive list of all components used in the development of CS2.0 with their price listings

Component	Quantity	Price	Purpose
Electronic Parts			
Hamamatsu C12880MA Micro Spectrometer	2 ($350 each)	$700	Spectrometers for CS2.0
TLP3556A Photorelay	1	$4.20	Turns sensors on and off
RockBlock 9603 Iridium Satellite Modem	1	$299.95	Satellite data transmission for CS2.0
RockBlock 9603 Accessory Cable	1	$4.95	Adapter cable to ESP32 interface
M1600HCT-P-SMA Iridium Passive Antenna	1	$64.95	Antenna for Iridium modem
SMA Male with Waterproof Ring & Bulkhead Antenna with Cable	1	$3.63	Antenna cable for Iridium modem
ESP32-S2 Saola Dev Kit	1	$14.50	Microcontroller for the system
DS3231 Real Time Clock	1	$3.80	RTC to control wake-up and sleep for ESP32
DIYHZ 1P2T SPFT Vertical Slide Switch	1	$0.09	Slide switch to toggle ESP32 between manual and autonomous mode
Voltaic 2W Solar Charger Kit	1	$5.99	Solar panel and battery
USB to Micro USB Cable	1	$4.99	Connects ESP32 to battery
SchmalzTech Breadboard ST-BB-470	2 ($8.99 each)	$17.98	Breadboard for system
Jumper Wire Kit	1	$11.99	Wires for breadboard
10 cm Jumper Wires Assorted Kit	1	$6.98	Jumper wires for RTC
40 cm Jumper Wires Assorted Kit	1	$11.99	Jumper wires for spectrometers
Mechanical Parts			
Waterproof Electrical Junction Box IP67	1	$19.99	Electronics housing
PVC 1-Gang Type FSE Box	1	$7.68	Sensor housing
TayMac 1-Gang Weatherproof Box Cover	1	$3.58	Cover for sensor housing
10 ft Schedule 40 PVC Conduit	1	$2.06	To connect sensor and electronic housings. Cut to 2 ft.
Schedule 40 PVC Bushing Connector Conduit Fitting	1	$0.60	Attaches PVC pipe to electronics housing
3/4-in Zinc-plated Steel Conduit Locknut	1	$0.39	Screws the bushing connector to the electronics housing

1-in Zinc-plated Standard Flat Washer	1	$0.78	Adds support to the inside of the electronics housing to prevent flex
1/2-inch Oversize Union Washer Flat Plumbing Slip Joint Washer Rubber	1	$0.30	Waterproofs the electronics box to pipe connection
3x30mm Round Aluminum Column Standoffs	4	$4.60	Standoff to support top layer in electronics box
3x16mm Thumb Screws	4	$5.20	To screw down top layer
4/40 Hex Machine Nuts	4	$0.24	Nuts to screw solar panel
M3x Screws	4	$0.94	Screws standoffs to mounting plate in electronics housing
Microscope Cover Glass Slips	2	$8.99 (not exact)	Cover glass were used as the lens for spectrometer housing
GE Advanced Silicone	1	$14.99 (not exact)	To seal solar panel and cable for waterproofing; to attach glass lens in sensor housing
Miscellaneous*	1	$20 (not exact)	TPU material used for 3D printed FOV adapter, PETG material, other 3D printed parts and foam pads used for battery
	**Total Cost**	**$1,300.33**	

*Gray card (external to the CS2.0 system) for radiometric calibration of the spectrometer can be obtained at a nominal price of $5: https://a.co/d/i3NDbEW

E. Heatproof Testing on Field

To test the heatproof design of the sensor and electronics housing, CS2.0 was deployed in autonomous mode for a week at Lake Herrick in Athens, Georgia. Ambient temperature and temperature inside the electronics and sensor housing box was recorded every half an hour. Tables 3 and 4 show the temperature variation from the most sunny and the most cloudy days of the week.

Table 3: Temperature Data from 3/31/23 (Red Text shows highest Box Temp)

Time	Box °C	Ambient °C	Outside Conditions
10:30 am	24.0	18.9	Cloudy
11:00 am	25.2	19.5	Cloudy
11:30 am	26.9	20.9	Cloudy
12:00 pm	29.8	22.2	Partially Sunny
12:30 pm	31.5	23.0	Partially Sunny
01:00 pm	32.6	23.7	Partially Sunny
01:30 pm	30.1	24.4	Cloudy
02:00 pm	31.5	24.6	Partially Sunny
02:30 pm	32.1	24.8	Partially Sunny
03:00 pm	28.9	24.3	Cloudy
03:30 pm	26.4	24.0	Cloudy
04:00 pm	32.1	23.9	Partially Sunny
04:30 pm	27.1	23.5	Cloudy
05:00 pm	31.5	23.2	Partially Sunny
05:30 pm	29.0	23.0	Cloudy
06:00 pm	27.0	22.7	Cloudy

Table 4: Temperature Data from 4/1/23 (Red Text shows highest Box Temp)

Time	Box °C	Ambient °C	Outside Conditions
01:00 pm	36.3	22.2	Sunny
01:30 pm	38.6	22.9	Sunny
02:00 pm	39.4	25.4	Sunny
02:30 pm	38.8	26.3	Sunny
03:00 pm	39.1	26.2	Sunny
03:30 pm	39.3	26.5	Sunny
04:00 pm	39.2	26.8	Sunny
04:30 pm	38.3	26.6	Sunny
05:00 pm	33.3	25.0	Sunny
05:30 pm	29.0	23.0	Cloudy
06:00 pm	27.0	22.7	Cloudy

F. Power Budget from Field Operation

Mode	Part	Current Consumption (mA)	Time in Mode (seconds/1 Day)	Charge Consumed (mA*s)
Idle				
	ESP32-S2	3		
	C12880mA (QTY: 2)	0		
	RockBLOCK Irdium 9603 M	0.1		
	TLP3556A Photorelay	0		
	Always on Battery	7		
	Total Idle:	*10.1*	*86097*	*869579.7*
Data Collection (Once Per Day)				
	ESP32-S2	32		
	C12880mA (QTY: 2)	40		
	RockBLOCK Irdium 9603 M	0.1		
	TLP3556A Photorelay	10		
	Always on Battery	7		
	Total Data Collection:	*89.1*	*3*	*267.3*
Transmission (1 Data Cycle Per Day)				
	ESP32-S2	32		
	C12880mA (QTY: 2)	0		
	RockBLOCK Irdium 9603 M	145		
	TLP3556A Photorelay	0		
	Always on Battery	7		
	Total Transmission:	*184*	*300*	*55200*
	Total:	*283.2*	*86400*	*925047*
	Consumption Per Day (mAh):	*10.71*	*Average Power Per Day (mW):*	53.53
**Battery**				
**Model**	**mAh**	**Battery Life (Days) No Recharge**	**Battery Draw Per Day (mAh)**	
Voltaic V25	6400	24.91	256.96	
**Solar Panel**				
**Model**	**Volts**	**Peak Watts**	**mA**	**Battery Return per Day (mAh)**
Voltaic P102	6	2.2	340	1020
			**Battery Return Per Day (mAh)**	1020
			**Battery Draw Per Day (mAh)**	256.96
			**Battery Gain Per Day (mAh):**	**763.04**

Figure 2: Actual power budget recorded after field-deployment of CS2.0

Table A: Calibration coefficients to convert CS2.0 AU to Radiance (390nm – 880nm)

Wavelength	Lw		Lc		Lsky	
	Coefficient	Bias	Coefficient	Bias	Coefficient	Bias
390	-0.377420664	6232.905	13.05778056	116470	3.007927386	43970.94
391	-0.31822665	5671.419	11.58347595	107223.1	2.659524231	40136.66
392	-0.283673801	5447.732	10.97939188	104127.6	2.527039034	38673.75
393	-0.252053223	5228.914	10.40399777	101064.3	2.399893116	37209.26
394	-0.20133233	5080.749	9.820725814	100274.6	2.224268735	36576.97
395	-0.171353458	5196.92	9.739188789	104370.7	2.189416713	37962.29
396	-0.164189121	5373.281	9.858000178	108557.6	2.232114083	39152.24
397	-0.166732543	5999.62	10.81811365	123296.9	2.465840795	43535.29
398	-0.169384947	6590.819	11.48004436	137701	2.658099038	47812.75
399	-0.181796642	7227.734	12.10525959	152174.5	2.873149956	51749.96
400	-0.192090515	8036.909	12.4003705	175655.9	3.131415331	56862.43
401	-0.222795853	8283.76	12.41530014	179658.6	3.197529273	57193.25
402	-0.251521545	8526.865	12.30716215	184576.2	3.260941638	57527.1
403	-0.269243666	8608.086	11.88870645	187942.1	3.262473675	56635.39
404	-0.286053748	8617.279	11.66481333	187241.1	3.245135617	55592.96
405	-0.300460512	8618.181	11.36599549	187125.8	3.225967583	54561.39
406	-0.306311257	8623.513	11.14471053	189422.6	3.242026289	53529.41
407	-0.318471205	8795.883	11.11447854	194319.4	3.322266214	53170.01
408	-0.334832259	8948.358	10.70657061	199365	3.378428783	52764.01
409	-0.353354029	9094.713	10.53986723	202886.8	3.447308776	52466.27
410	-0.363952303	9312.512	10.37199412	210647.1	3.555777653	52619.75
411	-0.380574994	9509.185	10.07485453	217455.6	3.645712163	52696.69
412	-0.411278136	9767.2	9.564034891	226160.6	3.744305954	52785.21
413	-0.42896204	9928.667	8.764538428	236511.5	3.822303668	52355.29
414	-0.448300993	10035.2	8.616180935	238928.4	3.86887533	51966.55
415	-0.466471134	10136.64	8.460274949	241408.4	3.915055861	51580.45
416	-0.470185205	10110.08	8.235706463	242072.7	3.922329896	50492.66
417	-0.486716481	10134.48	8.071849322	241899.8	3.924576815	49881.57
418	-0.502227969	10153.58	7.91077645	241703.4	3.925525397	49277.58
419	-0.510918898	10225.86	7.894340963	244660.1	3.974104264	48862
420	-0.537795881	10326.13	7.980960953	244842.3	4.029193663	48530.23
421	-0.563588116	10419.35	8.050386137	245160.7	4.081113403	48222.09
422	-0.572163781	10410.38	8.314467521	243910.7	4.103872234	47588.02
423	-0.577193174	10324.52	8.460137732	239919.9	4.078685852	46587.9
424	-0.599367043	10393.12	8.594059284	238817.6	4.091664849	46433.58
425	-0.626876918	10466.84	8.98596203	235843.4	4.082342477	46544.27
426	-0.631175062	10332.04	9.163530475	230655.8	4.000051568	45761.32
427	-0.619617344	10081.7	9.005153828	224372.8	3.894867082	44460.92
428	-0.606648042	9825.928	8.835191946	218178.2	3.788672614	43158.21

429	-0.561038858	9215.235	8.506050111	205399.7	3.590927477	40237.13
430	-0.570113136	9304.418	8.702257652	207105.9	3.659329187	40289.07
431	-0.578240853	9389.436	8.898709286	208813.3	3.727699605	40346.45
432	-0.587022747	9966.783	9.569912015	225863	4.033819808	42754.19
433	-0.59099272	10197.47	9.730694667	232794.4	4.122820593	43682.35
434	-0.594764439	10427.5	9.876147436	239839.5	4.211336079	44611.27
435	-0.589153659	10685.71	10.04438109	249348	4.343050994	45501.61
436	-0.571118906	10709.44	9.894504736	253717.8	4.386117311	45280.43
437	-0.557233199	10609.4	9.771882112	252965.8	4.372413169	44725.45
438	-0.542923125	10507.64	9.647375746	252227.2	4.358105065	44172.77
439	-0.522400982	10510.58	9.788509669	255020.1	4.409910718	43943.34
440	-0.521749439	10712.29	10.01203249	260974.7	4.492541929	44637.78
441	-0.520927516	10913.26	10.23126292	266958.7	4.574952564	45333.07
442	-0.51949674	11266.2	10.58432657	277186.3	4.718470005	46382.61
443	-0.504414425	11280.27	10.20394428	281081.2	4.749829451	45985.33
444	-0.488855282	11290.68	9.729485186	285712.5	4.78017559	45591.19
445	-0.478545415	11368.66	9.70159223	288072.4	4.810839507	45212.83
446	-0.4740744	11567.84	9.765876819	293293	4.891614618	45240.53
447	-0.474438534	11779.02	9.848005054	299231.4	4.988401611	45669.76
448	-0.471310601	11984.28	10.11585203	303570	5.058368711	46181.09
449	-0.473061576	12343.07	10.38235522	311328.8	5.156335673	46811.51
450	-0.465405798	12363.08	10.26797501	312393.1	5.164262001	46468.2
451	-0.457252401	12380.52	10.14650531	313514.1	5.171954069	46125.97
452	-0.440936068	12230.3	9.834662824	309820.6	5.086057474	44737.08
453	-0.425942747	12167.74	9.700054265	309697.4	5.071925859	44301.09
454	-0.410470641	12102.57	9.555039623	309656.1	5.057244874	43867.17
455	-0.401351345	12327.04	9.623289802	316461.5	5.126780786	44003.66
456	-0.385781828	12421	9.516204039	320779.7	5.145006571	43770.67
457	-0.370448274	12381.98	9.312389063	321981.8	5.126986026	43388.25
458	-0.355924119	12342.27	8.405580509	328947.4	5.137472448	42960.52
459	-0.333581403	12343.01	7.457138923	338435.7	5.166304464	42460.62
460	-0.321370479	12385.49	7.463701484	340866.3	5.177313335	42446.02
461	-0.309182285	12427.37	7.457393277	343398.4	5.187963512	42432.9
462	-0.286745006	12414.32	7.32763906	345810.5	5.169054216	42062.86
463	-0.264907712	12291.53	7.251180198	344374.4	5.156636772	41308.19
464	-0.242968012	12165.56	7.136884076	343240.3	5.142706396	40561.36
465	-0.20987202	11905.74	7.018596703	339121.3	5.057779797	39364.72
466	-0.181193955	11814.91	6.97083856	339549.6	5.033344198	38748.23
467	-0.163442148	11742.6	6.824896759	339959.3	5.011830765	38387.17
468	-0.140803081	11646.7	7.344805372	334995.3	4.998361506	37959.97
469	-0.107590211	11572.15	7.92239053	331292.3	4.998059578	37449.57
470	-0.084087516	11517.89	7.43942168	336310.2	5.015254389	37099.19
471	-0.060640716	11461.33	6.824216369	342389.4	5.031304863	36753.31

472	-0.0228028	11491.04	6.091305265	354531.4	5.091619996	36636.79
473	0.006325624	11377.26	6.404839267	352469.3	5.079065431	36294.27
474	0.0345321	11264.13	6.697515886	350561	5.064285541	35963.63
475	0.076608948	11101.56	5.699205033	359807.2	5.073742596	35308.75
476	0.120821996	10951.89	4.638147091	370443.4	5.106740199	34704.59
477	0.14685629	10891.21	3.494225006	381856.8	5.157457286	34425.86
478	0.166992877	10865.06	3.508715726	383930.9	5.193029803	34364.77
479	0.202498785	10802.74	3.387744969	387452.3	5.234968415	34160.77
480	0.224811677	10722.66	2.91331937	391760.7	5.243884077	34017.85
481	0.247264153	10641.49	2.434882702	396099.3	5.25265415	33875.82
482	0.281310916	10413.83	1.750260883	398328.8	5.198504097	33238.26
483	0.296318261	9932.445	1.238442726	386430.9	4.978863106	31706.02
484	0.303626211	9679.36	1.199278887	378508.5	4.871900901	30924.85
485	0.310483961	9423.961	2.061821542	363316.3	4.761384527	30100.72
486	0.326085331	9243.837	3.19144789	349661.6	4.69514689	29256.95
487	0.345323408	9344.968	2.523732382	361599.9	4.759563082	29574.61
488	0.364924721	9443.717	1.817634055	373838.6	4.823515938	29895.65
489	0.387452663	9581.807	0.678423501	390109.5	4.881568631	30107.83
490	0.399593228	9573.48	2.4121938	376687.1	4.894638663	29601.32
491	0.406058562	9559.578	4.33622045	361383.6	4.913089124	29254.48
492	0.422102663	9517.192	3.898860166	365229.1	4.911913489	29224.38
493	0.444286643	9599.35	3.548116795	372670.4	4.950969055	29335.84
494	0.462695101	9590.689	3.083261929	378245.4	4.962526336	29457.35
495	0.487639214	9545.159	1.982908827	388951.6	4.980634011	29570.57
496	0.506581422	9470.384	0.871690948	396188.6	4.943341397	29270.77
497	0.520091023	9391.494	0.909558726	393993.9	4.902396749	29201.71
498	0.534151127	9310.239	0.939182139	391866	4.860497239	29133.79
499	0.541415695	9202.687	1.038695418	385746.9	4.759547147	28687.99
500	0.561407503	9091.936	1.264532203	380660.7	4.649871362	28565.08
501	0.59073099	8995.64	1.500798416	378976.9	4.599094298	28826.42
502	0.609809094	8943.153	1.732874357	377321.7	4.568662511	28851.54
503	0.639145485	9052.038	1.94775519	381297.1	4.584773751	29156.23
504	0.672826777	8983.497	2.046391426	382554.2	4.603051281	29256.41
505	0.716914931	8871.242	2.341483544	382249.7	4.625394363	29463.83
506	0.768450706	8787.385	2.546938065	383148.3	4.659480213	29490.97
507	0.81161386	8683.309	3.501191505	375363.3	4.698484792	29231.03
508	0.858555075	8574.38	4.274737332	370790.3	4.756700755	29318.32
509	0.894503379	8522.242	4.607272561	369752.1	4.801373154	29398.57
510	0.934355743	8491.387	4.78717513	369000.4	4.838885001	29311.02
511	0.965416311	8391.752	4.956806642	366556.6	4.84314433	29264.88
512	1.028313484	8159.011	5.30977401	362755.1	4.901098016	29156.69
513	1.08782087	7873.258	5.44359426	356262.5	4.894266847	28640.26
514	1.105477981	7558.428	5.716809989	343145.6	4.775013813	27638.18

515	1.13400623	7318.749	6.079371063	335269.8	4.732612145	27163.1
516	1.163167526	7076.118	6.398602169	327735.5	4.689612548	26688.43
517	1.243585779	6778.348	6.582445157	325615.8	4.688048902	26385.91
518	1.345912107	6514.395	6.819358343	329297.6	4.765893193	26670.28
519	1.435319642	6261.497	6.715855534	335549.2	4.807224446	26966.42
520	1.538752748	6134.963	6.596439842	345698	4.880558642	27378.93
521	1.612462805	6057.544	6.674854833	351075.1	4.92335125	27475.01
522	1.667189485	5836.344	6.692514482	352003.2	4.917310248	27415.03
523	1.723524666	5603.051	6.709643158	352935.3	4.911227461	27355.27
524	1.776345225	5236.417	6.222701321	352634.1	4.8117601	26940.88
525	1.845250212	4786.314	5.785045631	354475.1	4.758290233	26791.76
526	1.923165914	4308.963	5.518612315	355039	4.741480185	26529.82
527	2.013407554	4075.983	5.385699512	360705.2	4.786341638	26546.68
528	2.090005936	4128.909	5.582902695	369058.1	4.86009208	27017.21
529	2.134360833	3907.558	5.641160228	369080.2	4.835108604	26989.32
530	2.179512359	3677.91	5.696349273	369125	4.8102764	26961.26
531	2.226649618	3417.387	5.70994434	365959.1	4.752289198	26657.57
532	2.317453051	2930.463	5.408945712	368256.9	4.75953583	26569.83
533	2.378103378	2642.504	5.512440857	367327.8	4.764653515	26442.88
534	2.474321999	2353.017	5.058879911	373762.8	4.825484123	26277.21
535	2.543317505	2131.993	5.020003641	374888.7	4.83825812	26065.15
536	2.644161578	1558.024	4.844611057	374629.3	4.816544097	25938.46
537	2.741960863	999.7602	4.670722273	374354.8	4.794489527	25812.02
538	2.79454347	581.0849	4.547480079	370844.8	4.714056832	25348.65
539	2.852567872	136.7766	4.505480326	369389.5	4.675762403	25151.64
540	2.898274719	-118.227	4.719176434	365940.6	4.653295555	24937.37
541	2.969540751	-355.254	5.00258842	364163.4	4.648504731	24731.95
542	3.070338515	-630.641	5.254752424	365827.7	4.689557599	24733.43
543	3.132030717	-892.683	5.342540462	366584.5	4.695803266	24825.28
544	3.188960516	-1128.4	5.431283472	367334.5	4.701925032	24917.82
545	3.249348974	-1384.45	5.457154093	367421.1	4.708061695	24737.18
546	3.295569106	-1617.07	5.540596949	366034.4	4.702153029	24493.76
547	3.341819485	-1872.14	5.29750825	367911.5	4.721238918	24307.62
548	3.386784061	-2120.55	5.055634152	369775	4.740364026	24121.51
549	3.420802673	-2134.36	5.188698846	369299.7	4.739897331	24006.54
550	3.431808409	-2080.99	5.281424157	368661.8	4.745262427	23962.68
551	3.442952045	-2027.95	5.365986553	368089.2	4.750556183	23919.36
552	3.478898694	-2135.44	5.329259745	368929.6	4.771079795	23603.31
553	3.503487409	-2192.07	5.324037436	369230.3	4.789498047	23284.8
554	3.494392461	-2004	5.789458003	364866	4.807279547	23159.85
555	3.484994029	-1814.88	6.207407913	360880.7	4.825071754	23036
556	3.438959308	-1650.58	6.339471418	355228.9	4.779349601	22566.17
557	3.402621494	-1474.06	6.552041104	351475.4	4.766770541	22351.58

558	3.366792687	-1301.11	6.74935095	347839.3	4.754060924	22137.11
559	3.340800089	-1068.58	6.874995903	345377.9	4.762472989	21826.55
560	3.336897685	-837.594	7.072798458	345290.9	4.797772299	21698.05
561	3.336207696	-645.177	7.537037711	342558.6	4.825384809	21694.39
562	3.334627101	-447.85	7.967191725	340099.5	4.853075763	21692.07
563	3.327857643	-263.863	8.192534821	338879.1	4.873303124	21509.52
564	3.305097142	-62.8356	8.415260343	336451.8	4.870148926	21273.41
565	3.284272287	120.7501	8.619922527	334287.3	4.870663485	21151.75
566	3.271408045	281.1079	9.096940005	330010.4	4.863496211	21050.97
567	3.244206503	432.6905	9.534094352	324612.1	4.799973229	20829.13
568	3.204705546	736.5909	9.886859293	320966.1	4.779440306	20789.57
569	3.1629659	1048.402	10.21383404	317530.7	4.758254737	20750.77
570	3.096189382	1333.867	10.62045194	313629	4.721167522	20505.87
571	3.035318136	1647.76	11.03703382	311194.5	4.712807011	20321.95
572	2.998917265	1940.997	11.03795114	312382.3	4.716966423	20349.57
573	2.961788688	2235.976	11.03744651	313581	4.720946851	20377.88
574	2.903825567	2540.175	10.9703473	313950.2	4.706024639	20237.05
575	2.827458473	2902.287	10.97330146	313338.1	4.694533575	20139.54
576	2.751265333	3262.321	10.97202228	312758.4	4.682972596	20042.18
577	2.699030566	3458.834	10.69895351	314270.4	4.696213845	19740.28
578	2.654975216	3656.351	10.45350586	316804	4.732229697	19498.64
579	2.588908155	4035.465	10.55611068	316991.7	4.735135282	19557.98
580	2.524116245	4405.976	10.64427897	317286.8	4.737943207	19617.68
581	2.523417318	4489.076	10.8142894	319090.5	4.813729759	19521.95
582	2.510635358	4557.967	10.94518851	319760.2	4.863924546	19375.94
583	2.491467533	4646.637	11.07467099	318801.1	4.879451144	19260.31
584	2.494297745	4711.079	11.62366275	314758.4	4.914354619	19148.61
585	2.457893411	4702.903	12.14655363	306759.2	4.828603205	18886.81
586	2.388139163	4925.634	12.75338903	297798.5	4.725314865	18797.47
587	2.315690877	5152.333	13.32937049	289078.9	4.62053177	18706.45
588	2.215527447	5169.152	13.97934575	275048.9	4.348105729	18392.23
589	2.136563051	5213.018	14.63991381	264464.7	4.159296006	18160.07
590	2.065297166	5586.33	15.03917936	262184.4	4.14445095	18197.05
591	1.989275329	5973.171	15.42906007	259984.9	4.129106442	18234.88
592	1.914291912	6243.589	15.38410087	262449.7	4.13448641	18210.83
593	1.837605901	6560.898	15.22196282	264449.6	4.147946313	18175.18
594	1.762499774	6867.637	15.05652471	266477.4	4.161380555	18139.67
595	1.668940878	7185.082	14.76352198	270793.2	4.233753803	17948.61
596	1.583777192	7418.357	14.34402517	275735.8	4.329994881	17690.48
597	1.49441843	7776.585	13.96120711	279415.3	4.39927276	17571.77
598	1.410643575	8101.153	13.58560227	283037	4.469127155	17453.45
599	1.356143943	8093.584	12.93850379	288328.3	4.561258281	17160.32
600	1.316703789	8052.255	12.35002694	293722.9	4.649557742	16914.68

601	1.289503876	8094.868	12.01913505	296967	4.688572353	16790.75
602	1.266354412	8119.277	11.68593792	300227.8	4.727645744	16666.27
603	1.292943945	7912.392	11.61663436	303038.7	4.795302199	16590.25
604	1.289614307	7899.373	11.58482182	303977.6	4.817073228	16571.03
605	1.287658358	7880.472	11.55159739	304927.5	4.838833266	16552.24
606	1.294612754	7725.054	11.44667144	305273.7	4.848148819	16366.76
607	1.304248836	7560.417	11.34811447	305052.6	4.842251187	16176.14
608	1.30915988	7484.236	11.03959222	306566.5	4.824658172	16127.32
609	1.314950035	7404.624	10.72629194	308108.9	4.806878671	16078.75
610	1.340578097	7180.74	10.84199598	305305.9	4.794186297	15911.69
611	1.367278583	6960.794	10.95632648	302660.2	4.781280435	15755.33
612	1.366597012	6891.186	10.83886727	302508.2	4.753004485	15684.18
613	1.366744522	6818.209	10.72036939	302366.4	4.724745461	15613.33
614	1.38538355	6593.762	10.41384704	302391.2	4.673207952	15537.08
615	1.403345015	6402.92	10.11822933	303221.2	4.632415126	15500.19
616	1.425453849	6309.909	10.16142452	303351.2	4.645432873	15472.62
617	1.44777538	6216.883	10.20066136	303512.3	4.658447581	15445.25
618	1.494173388	6039.807	10.25772953	305063.4	4.68299133	15421.4
619	1.488411104	6055.545	10.1909809	306162.6	4.674618893	15420.48
620	1.483004419	6069.802	10.12306549	307271.2	4.666139266	15419.86
621	1.502586354	5942.39	10.11015795	306518.5	4.647881044	15378.26
622	1.511059306	5839.471	10.07943919	305006.7	4.61618678	15359.44
623	1.519668655	5763.187	10.29289936	302225.2	4.612564831	15262.79
624	1.52835181	5686.865	10.49760928	299514.6	4.608632678	15166.97
625	1.504924093	5680.195	10.61406219	295859.1	4.545277009	15151.98
626	1.479733286	5701.879	10.78598504	292586.3	4.48214211	15131.04
627	1.48597457	5657.725	11.03422487	289755.5	4.45262453	15115.73
628	1.492193528	5613.972	11.26800322	287038.7	4.422641902	15100.71
629	1.50617505	5482.126	11.23535708	286383	4.39031478	14897.23
630	1.520440291	5403.557	11.18500177	286705.4	4.387253651	14795.74
631	1.522481639	5388.205	11.28725864	285719.1	4.384638068	14757
632	1.529331417	5370.272	11.39195232	285607.9	4.400924477	14715.34
633	1.548751809	5313.472	11.27418575	287597.8	4.431292562	14675.36
634	1.565128744	5277.097	11.16826283	289302.6	4.453691853	14657.65
635	1.587723598	5200.731	11.20516584	289809.1	4.475522454	14601.9
636	1.608934827	5141.915	11.22048203	290940.3	4.508984008	14472.94
637	1.606905126	5197.299	11.26959652	291592.7	4.53048823	14491.04
638	1.60166562	5232.47	11.30722308	291130	4.530124129	14548.59
639	1.596516567	5267.194	11.34309416	290680.6	4.52966165	14606.4
640	1.602436254	5250.965	11.48971766	289219.3	4.536719089	14599.51
641	1.607708905	5222.869	11.65711669	287379.3	4.529743925	14544.57
642	1.621170483	5197.931	12.02049384	284336.3	4.534165566	14523.7
643	1.635264865	5171.449	12.3668955	281438.2	4.538245098	14503.89

644	1.592069479	5257.775	12.63753268	277178.6	4.439513072	14426.74
645	1.566625884	5290.354	12.88271687	273449.5	4.366182855	14360.29
646	1.542648469	5334.175	13.44898524	267504.8	4.30286659	14266.81
647	1.489479311	5409.913	14.089479	259168.3	4.178855064	14056.77
648	1.449665965	5505.425	14.32372968	255307.2	4.120876818	13954.83
649	1.445449999	5556.2	14.45466952	254649.2	4.136426158	13998.53
650	1.433730956	5627.935	14.64652385	253528.7	4.146906563	14029.09
651	1.426707939	5688.135	14.78223603	253675.9	4.17965333	14149.2
652	1.412262227	5722.666	14.84186758	252985.8	4.199367921	14162.63
653	1.390476292	5736.903	14.86266958	250658.6	4.18906199	14059.73
654	1.353924308	5808.465	14.78742672	248598.8	4.196653761	13940.27
655	1.313173817	5730.737	14.50964875	244914.9	4.177227995	13640.29
656	1.298826408	5607.906	14.13093748	246234.3	4.194184199	13432.17
657	1.297471772	5604.481	13.9654812	249661.8	4.242622393	13403.94
658	1.298486753	5605.873	13.87872639	252666.9	4.287283804	13430.03
659	1.360672729	5415.919	13.80506205	259619.1	4.407438019	13447.31
660	1.374048824	5374.198	13.65403308	263096.9	4.463417804	13428.41
661	1.388717989	5328.829	13.50408098	266571.3	4.519141095	13410.61
662	1.445606318	5082.621	13.37742942	271062.1	4.613621165	13338.07
663	1.494400618	4834.538	13.234578	274248.9	4.678010422	13360.24
664	1.517713088	4746.144	13.27711778	274871.2	4.709652933	13433.85
665	1.541880118	4656.743	13.31718871	275513.7	4.741517817	13507.97
666	1.608183325	4359.15	13.45673762	275352.6	4.779051646	13487.83
667	1.678422186	4025.024	13.55522736	274747.5	4.804390696	13334.03
668	1.722198125	3880.494	13.81856887	273559.8	4.835279694	13256.65
669	1.76678076	3736.607	14.07302427	272466.3	4.866177622	13180.17
670	1.826667155	3472.01	14.09283675	271702.2	4.877326222	12998.93
671	1.878550443	3246.471	14.10018233	270953.8	4.892277428	12731.9
672	1.904833731	3140.14	14.2624179	269577.6	4.91531373	12515.33
673	1.931458212	3033.438	14.41266427	268290.3	4.9384233	12298.72
674	1.990219277	2815.848	14.45699258	267589.4	4.955242702	11969.62
675	2.045130442	2634.584	14.49332158	267127.1	4.96987079	11671.26
676	2.078188509	2549.906	14.59286723	266562.2	4.977259013	11577.52
677	2.111640684	2465.296	14.6910255	266015.2	4.984492485	11484.37
678	2.148190268	2333.931	14.76966355	265064.9	4.989666204	11190.84
679	2.16067582	2280.796	14.82703589	263631.2	4.982362697	10871
680	2.177197548	2297.545	14.97104847	262886	5.017114711	10748.9
681	2.194118965	2314.215	15.10987239	262196.9	5.052173994	10628.13
682	2.173201634	2374.066	15.33052474	257869.4	4.997012432	10302.18
683	2.146313461	2397.53	15.53858229	252128.3	4.923226119	10043.3
684	2.126011971	2376.575	15.67240978	246427.8	4.835681718	9768.549
685	1.972557396	2522.859	15.55068429	231913.2	4.523418268	9182.67
686	1.868728258	2680.244	15.82687868	219341.2	4.333282784	8709.758

687	1.851147153	2755.592	16.340023	215925.2	4.308172067	8579.288
688	1.838404393	2817.8	16.86898141	212497.8	4.281062228	8452.571
689	1.734450492	3308.293	17.77481645	211354.9	4.260987279	8573.795
690	1.59734852	3887.018	19.2391564	207233	4.174635819	8867.405
691	1.557191014	4051.937	19.9049578	203949.5	4.092695899	9016.607
692	1.535558324	4168.178	20.57676564	200613.1	4.011118491	9165.017
693	1.475808541	4463.301	21.54685173	197025.9	3.942628081	9297.362
694	1.513157426	4531.729	21.75351518	199508	4.013792061	9285.314
695	1.593823095	4419.428	21.84349929	201442	4.077574784	9292.044
696	1.689078507	4271.184	21.92814502	203413.5	4.141810194	9299.784
697	1.736050004	4197.167	22.02839344	203503.7	4.15265757	9232.175
698	1.770755703	4066.048	22.43538198	199800.6	4.065278786	9220.154
699	1.819273243	3912.684	22.55415929	198574.9	4.035284358	9191.242
700	1.872158214	3748.719	22.66986997	197371.8	4.004823896	9163.227
701	1.876108787	3722.874	22.48731709	198648.8	4.017634415	9146.456
702	1.902969367	3664.006	22.14623322	202700.2	4.100735436	9114.224
703	1.946290998	3566.128	22.16956042	204823.4	4.188829425	8973.084
704	1.916103409	3664.716	21.98289678	207519.4	4.229961065	8979.631
705	1.88285323	3716.37	21.61216692	212016.8	4.305353086	8955.476
706	1.820475774	3800.77	21.17794258	216057	4.387960634	8879.348
707	1.757163958	3925.872	20.95051057	218162.3	4.436027724	8837.759
708	1.698283096	4038.342	20.8887779	219664.8	4.485038185	8772.166
709	1.619409557	4094.266	20.68562918	221888.6	4.545832345	8669.111
710	1.529478353	4161.527	20.76682175	222375	4.594640847	8602.783
711	1.445465746	4287.674	21.08689521	220944.8	4.601542055	8584.401
712	1.379100066	4375.44	21.86506363	218250.9	4.627693957	8524.461
713	1.213921418	4451.006	23.48669242	206055.2	4.402617891	8576.043
714	0.99370524	4477.67	26.32885598	180545.6	3.858961206	8761.554
715	0.844321331	4528.459	28.70762799	161687.4	3.483911365	8882.455
716	0.635844144	4718.75	31.86756083	141439.7	3.021814006	9225.566
717	0.403511661	4633.333	35.16026409	110127.5	2.316486468	9367.329
718	0.260780319	4581.134	36.92794869	97224.7	2.00250476	9451.62
719	0.186702823	4666.925	37.55604387	98353.55	2.013395011	9510.775
720	0.111536353	4749.85	38.1671637	99651.14	2.022125831	9574.218
721	0.036297153	4806.211	38.048094	105519	2.111411046	9628.163
722	-0.060021186	4834.179	38.81510754	103182.3	2.03154665	9724.769
723	-0.148686079	4901.52	39.33326941	99499.99	1.960386129	9727.681
724	-0.2328904	4959.065	39.8467502	95841.07	1.888609468	9732.247
725	-0.393739957	5066.55	40.72008126	90703.67	1.764020123	9792.422
726	-0.454556551	5005.858	40.97508749	89251.23	1.72781465	9770.894
727	-0.498845148	5008.828	41.01701593	89840.14	1.74739081	9739.386
728	-0.544590285	5019.117	40.67991662	90786.25	1.745436581	9732.991
729	-0.564101556	4947.947	39.81259883	97393.47	1.86310015	9685.699

730	-0.545546926	4849.358	38.08626553	111386.1	2.167135776	9537.758
731	-0.53752465	4824.499	36.72807486	121664.5	2.396446935	9418.626
732	-0.538259162	4830.018	34.71991999	132952.1	2.599767904	9265.135
733	-0.480715962	4712.898	32.54014872	150836.1	3.011195083	9024.632
734	-0.437481682	4604.594	30.77435445	164251.9	3.324116611	8805.301
735	-0.424676838	4558.9	29.89662832	169992.1	3.435442542	8727.575
736	-0.410545798	4510.987	28.99171873	175851.9	3.546800198	8651.092
737	-0.375706038	4420.235	27.73761282	183865.2	3.740565134	8421.568
738	-0.338861826	4334.516	26.43687561	191496.8	3.94198056	8148.794
739	-0.308933852	4270.197	25.5354856	197460.1	4.06996412	8066.425
740	-0.281993754	4217.883	25.0014854	201359.5	4.170864855	7969.589
741	-0.237598807	4141.372	24.1426224	208075.7	4.360615596	7791.812
742	-0.198135436	4078.543	23.48983454	213907.5	4.545515372	7599.933
743	-0.178528173	4044.626	23.20344969	216419.1	4.622677121	7501.287
744	-0.160225111	4010.536	23.12742653	218623.6	4.699205402	7477.278
745	-0.133058386	3957.012	22.97311613	221670.5	4.806614093	7399.626
746	-0.103148943	3892.526	22.62579484	223325.9	4.848730305	7204.339
747	-0.087275311	3842.994	22.50507433	224306.4	4.899263022	7084.083
748	-0.07847183	3812.951	22.47755075	224656	4.925973221	7020.67
749	-0.069211308	3782.077	22.63526147	224254.5	4.919277117	7025.237
750	-0.057485295	3737.61	22.77459476	223744.9	4.90906386	7000.293
751	-0.03405792	3675.572	23.09852743	223090.6	4.955565697	6826.587
752	-0.027142588	3653.749	23.31033366	223333.5	4.999056355	6790.021
753	-0.020039762	3631.804	23.51966302	223612	5.042324796	6755.196
754	-0.009996406	3597.185	24.05604376	222889.9	5.097690786	6656.695
755	-0.005230737	3537.746	24.40363298	219708.5	5.080828008	6521.941
756	0.040339664	3356.877	26.7000221	208628.5	4.912852148	6453.518
757	0.059808695	3222.246	27.44857598	200319.4	4.715236375	6431.642
758	0.069503807	2812.087	26.23241366	172613	3.978825447	5931.262
759	0.060212935	2339.067	24.44204458	137474	3.064516244	5179.169
760	0.064282831	2144.041	24.82105991	122469.2	2.68318876	4941.178
761	0.072384446	1944	25.49833444	108009.1	2.25711579	4824.596
762	0.066723428	1793.191	25.61963792	96539.97	1.898978414	4666.673
763	0.083872201	1809.698	27.1612321	98736	1.924284694	4823.081
764	0.108365407	1974.938	28.20775686	113906.8	2.297418069	5133.205
765	0.123423808	2097.189	28.26999988	125291.6	2.565020398	5337.086
766	0.118454403	2252.345	26.27665025	140919.9	2.887388137	5451.014
767	0.148209167	2498.272	26.83484953	163287.3	3.484880489	5776.253
768	0.171432549	2696.507	26.89217169	181496.6	3.973076985	5997.094
769	0.171970712	2777.773	26.3108215	189084	4.147462177	6036.053
770	0.172030571	2859.842	25.75342549	196646.8	4.31937457	6076.131
771	0.187161105	2939.414	25.34765052	205906.8	4.568541602	6152.671
772	0.198685334	2974.738	25.24427401	210085.8	4.674311871	6146.696

773	0.19899267	2987.178	25.05969021	211231.3	4.689817719	6116.29
774	0.222509321	2964.615	25.13163122	213254.2	4.799036495	6036.985
775	0.236111437	2960.991	25.35261016	214224	4.85167066	6021.324
776	0.246745507	2953.774	25.52534219	214500.9	4.870647443	6012.887
777	0.265687723	2923.316	25.54494095	214338.2	4.895660463	5900.98
778	0.278991426	2900.572	25.59567523	214141.2	4.912423348	5825.258
779	0.297447684	2874.197	25.9156051	214112.8	4.834812065	5993.341
780	0.310423064	2855.052	25.88924137	214117.8	4.859829761	5899.564
781	0.327626431	2825.985	25.8715157	213825.6	4.87596874	5803.637
782	0.333938257	2810.149	26.40381971	212314.1	4.854847841	5735.146
783	0.339897455	2803.203	26.77924634	211467.5	4.820483331	5815.328
784	0.341777128	2797.896	27.30956786	209328.9	4.714949969	5934.176
785	0.330271738	2795.94	28.41700968	204376.7	4.571099495	5958.705
786	0.310803654	2802.75	29.56148386	198762.6	4.366208075	6097.077
787	0.302474868	2802.041	30.51138376	195042.1	4.256196934	6179.178
788	0.289760272	2807.854	31.13132332	192030.8	4.149382488	6253.272
789	0.271075502	2807.527	31.89380309	186876.1	3.97159722	6328.562
790	0.289798763	2755.038	32.664405	182951.2	3.880841878	6280.933
791	0.301615619	2733.648	32.87180694	182148.6	3.886080235	6244.198
792	0.313588597	2712.191	33.07705842	181363.2	3.890865398	6208.275
793	0.352629451	2652.506	33.28042781	181313.5	3.906717868	6150.687
794	0.371734727	2622.225	33.43963549	180210.9	3.862894457	6123.789
795	0.380785277	2613.641	33.92180847	178634.3	3.792591204	6149.13
796	0.399621967	2595.954	34.50301902	178911.9	3.791223446	6209.354
797	0.408569328	2582.496	34.95030055	177690.6	3.742129035	6223.925
798	0.415823783	2572.128	35.40369267	176042.9	3.666513184	6238.841
799	0.420456989	2567.942	36.18392706	174663.7	3.581305956	6346.996
800	0.4207835	2555.634	36.41001712	172661.8	3.495954898	6355.829
801	0.426546801	2542.733	36.49262686	172010.8	3.466035336	6354.447
802	0.431773034	2526.354	36.36787973	171912.1	3.452297783	6289.088
803	0.457091261	2492.646	36.43131247	172194	3.482736032	6223.173
804	0.49012981	2456.42	36.24889811	173928.5	3.55049149	6149.441
805	0.519803644	2418.163	36.5729787	174981.4	3.574781252	6138.303
806	0.531047645	2404.407	36.65900076	175185.3	3.605576263	6083.367
807	0.537401423	2385.659	36.7894249	174306.4	3.596524723	6028.838
808	0.530287748	2380.792	38.38165347	170301.7	3.431361245	6247.002
809	0.468919083	2410.729	40.71117981	160209.7	3.128530324	6423.86
810	0.41621095	2442.424	42.97987634	151252.2	2.886379279	6575.505
811	0.380935736	2460.271	46.0919549	143759.9	2.593299757	6900.705
812	0.263040273	2507.755	50.60820123	123791.6	2.057452352	7209.766
813	0.13659891	2538.952	55.59410623	100715.7	1.490790761	7467.268
814	0.022677158	2552.147	62.16809446	75121.54	0.964826255	7666.432
815	-0.018374105	2550.143	63.83610382	70022.88	0.693382813	7865.168

816	-0.05646504	2542.971	65.08840607	63840.72	0.524733658	7895.222
817	-0.081084096	2505.592	65.59157619	59868.67	0.421519322	7830.894
818	-0.046265738	2436.637	64.47388524	64868.35	0.459577905	7785.851
819	-0.004310919	2369.328	62.0825818	73400.97	0.560831925	7706.489
820	0.012724172	2335.42	60.5952039	78432.89	0.568173896	7716.396
821	0.020568804	2314.824	57.5065099	84690.49	0.686597349	7537.865
822	0.032918433	2264.903	55.97269886	88657.32	0.793804008	7365.627
823	0.059257745	2213.778	54.16460326	95744.51	0.936321338	7248.717
824	0.091826154	2165.517	51.58271302	106929.6	1.038128263	7260.78
825	0.099740076	2144.256	49.92097275	112471.8	1.116948451	7185.329
826	0.109855716	2120.888	48.71113076	117187.1	1.138801972	7210.005
827	0.118477609	2066.166	47.51803157	121219.2	1.161436115	7196.406
828	0.12751722	2001.503	44.26275501	129050.4	1.232128842	7074.522
829	0.136324469	1938.274	42.6721622	133018.3	1.272366927	7003.581
830	0.142222974	1908.536	41.21790602	136870.8	1.313983602	6949.521
831	0.151619653	1876.754	38.70354257	144176.8	1.371497611	6899.998
832	0.172292435	1818.734	35.37168304	153332.5	1.547891742	6694.042
833	0.203907985	1765.869	32.72638491	162828	1.739230278	6543.466
834	0.244785551	1713.435	29.96516342	173449.1	1.957855661	6392.486
835	0.259115804	1700.621	26.50288463	183678.2	2.069039316	6323.69
836	0.282423885	1668.162	24.11588665	190892.4	2.172202334	6215.054
837	0.324775225	1625.935	22.43899524	198864.9	2.427942012	6021.498
838	0.359872376	1592.416	20.65887577	206309.4	2.685367335	5797.178
839	0.400838488	1543.462	17.58308274	216351	2.896280291	5598.427
840	0.412737486	1526.76	16.58675084	219445	2.969346413	5509.736
841	0.424523375	1510.082	15.63223661	222450.9	3.041777336	5421.074
842	0.469415468	1453.634	12.75527376	230749.7	3.267218426	5190.525
843	0.504563119	1395.499	10.80254295	236232.3	3.326751601	5098.281
844	0.529251487	1361.793	10.42037978	238144.7	3.426613606	5010.822
845	0.543224537	1342.793	9.627421028	240219.5	3.439444717	5016.55
846	0.556640757	1329.504	7.344122303	245868.6	3.528246054	4942.477
847	0.567975597	1292.651	5.112923214	249840.5	3.549609648	4828.175
848	0.588913213	1249.105	3.464942713	251559.7	3.530616382	4835.098
849	0.586619701	1225.318	2.403036961	251639.7	3.547000074	4693.695
850	0.587960582	1187.888	1.073581616	251580.8	3.524842005	4578.151
851	0.592286543	1156.399	-0.849353776	253856.5	3.411448256	4627.137
852	0.603447678	1133.639	-2.474413937	255391.7	3.380857555	4662.204
853	0.596906481	1106.471	-2.146702245	251380.7	3.349346506	4546.92
854	0.600458993	1084.046	-1.695733052	249702.5	3.383596603	4430.297
855	0.618855857	1067.697	-3.074423148	254975.8	3.466168251	4399.429
856	0.626305567	1067.131	-3.81749288	258127.6	3.51880871	4383.597
857	0.633883643	1061.335	-3.902802134	259945	3.536287399	4391.12
858	0.655635833	1037.15	-3.969317947	262794.1	3.594169596	4371.342

859	0.66531555	1040.44	-4.342072169	265177.5	3.642694514	4412.814
860	0.671241957	1035.148	-4.172710748	265854.6	3.691587111	4371.378
861	0.676043071	1019.093	-5.829783005	269280.3	3.707469599	4306.885
862	0.678587352	1008.161	-6.480165229	269506	3.673728056	4339.799
863	0.681052142	997.3743	-7.125404291	269702.8	3.639754407	4372.603
864	0.695850575	939.7268	-8.026063476	267907.5	3.592758369	4263.24
865	0.695511607	913.2515	-9.792132225	267735.5	3.516780897	4249.948
866	0.69337276	903.8066	-9.793257958	266066.4	3.537213725	4188.686
867	0.697558932	888.8774	-10.31588539	267520	3.546129735	4143.582
868	0.701748781	873.8669	-10.83503907	268971	3.553670968	4100.31
869	0.699243735	888.5477	-12.31013015	273357.5	3.541444174	4203.705
870	0.712594142	873.0847	-14.71575363	279375.7	3.451457299	4381.766
871	0.718073704	857.4306	-14.48617011	279549.7	3.590003388	4171.415
872	0.728794813	842.0541	-14.74903533	279899.7	3.535271087	4263.065
873	0.733339411	834.9826	-14.88398637	279761	3.49007952	4343.357
874	0.737143664	818.2899	-15.9494114	281605.9	3.455462362	4349.28
875	0.737412954	809.4464	-17.80930207	284624.5	3.416677621	4374.705
876	0.739301965	799.2059	-19.12103652	286469.5	3.393120564	4389.805
877	0.725313318	797.1601	-19.57454254	286791.9	3.442097162	4235.108
878	0.73059549	764.0284	-20.2215329	287149.6	3.465135183	4107.279
879	0.733646633	766.2975	-20.48495491	286585.2	3.409954243	4239.594
880	0.734283219	756.2713	-20.99524379	286887	3.390746412	4238.763
